# Machine learning-based prediction of the post-thrombotic syndrome: Model development and validation study

**DOI:** 10.3389/fcvm.2022.990788

**Published:** 2022-09-16

**Authors:** Tao Yu, Runnan Shen, Guochang You, Lin Lv, Shimao Kang, Xiaoyan Wang, Jiatang Xu, Dongxi Zhu, Zuqi Xia, Junmeng Zheng, Kai Huang

**Affiliations:** ^1^Department of Emergency, Sun Yat-sen Memorial Hospital, Sun Yat-sen University, Guangzhou, China; ^2^Zhongshan School of Medicine, Sun Yat-sen University, Guangzhou, China; ^3^Department of Cardiovascular Surgery, Sun Yat-sen Memorial Hospital, Sun Yat-sen University, Guangzhou, China

**Keywords:** deep vein thrombosis, machine learning, post-thrombotic syndrome, prognosis, endovascular

## Abstract

**Background:**

Prevention is highly involved in reducing the incidence of post-thrombotic syndrome (PTS). We aimed to develop accurate models with machine learning (ML) algorithms to predict whether PTS would occur within 24 months.

**Materials and methods:**

The clinical data used for model building were obtained from the Acute Venous Thrombosis: Thrombus Removal with Adjunctive Catheter-Directed Thrombolysis study and the external validation cohort was acquired from the Sun Yat-sen Memorial Hospital in China. The main outcome was defined as the occurrence of PTS events (Villalta score ≥5). Twenty-three clinical variables were included, and four ML algorithms were applied to build the models. For discrimination and calibration, F scores were used to evaluate the prediction ability of the models. The external validation cohort was divided into ten groups based on the risk estimate deciles to identify the hazard threshold.

**Results:**

In total, 555 patients with deep vein thrombosis (DVT) were included to build models using ML algorithms, and the models were further validated in a Chinese cohort comprising 117 patients. When predicting PTS within 2 years after acute DVT, logistic regression based on gradient descent and L1 regularization got the highest area under the curve (AUC) of 0.83 (95% CI:0.76–0.89) in external validation. When considering model performance in both the derivation and external validation cohorts, the eXtreme gradient boosting and gradient boosting decision tree models had similar results and presented better stability and generalization. The external validation cohort was divided into low, intermediate, and high-risk groups with the prediction probability of 0.3 and 0.4 as critical points.

**Conclusion:**

Machine learning models built for PTS had accurate prediction ability and stable generalization, which can further facilitate clinical decision-making, with potentially important implications for selecting patients who will benefit from endovascular surgery.

## Introduction

Post-thrombotic syndrome (PTS) is a common sequela of deep vein thrombosis (DVT), which is caused by chronic venous insufficiency (CVI), secondary to prior DVT, and can affect up to 50% of patients with proximal DVT within 2 years (1, 2). However, its pathophysiology remains unclear. Nonetheless, similar to other forms of CVI, PTS is mostly caused by venous hypertension, which is attributed to an irreversibly fibrosed vein wall, valvular damage, or residual venous obstruction after acute DVT (3). The clinical symptoms of PTS can manifest as heaviness, pain, edema, pruritus, and spasticity of the lower extremities, which are often aggravated during standing or walking and relieved while resting or lying down (4). PTS heavily affects the quality of life and has an effect comparable to that of heart failure or diabetes mellitus (5), which could cost an estimated annual direct cost of US $200 million and an annual loss of 2 million workdays in the United States (6, 7). Existing treatment options for PTS remain limited despite its severe harm to health and a high socioeconomic impact (8). Preventative interventions remain a key measure to reduce the incidence, impact on quality of life, and treatment cost of PTS.

Preventing PTS remains a huge challenge as symptoms of PTS change gradually during chronic progression. Many previous studies have identified predictors that may help in the risk stratification of patients with PTS. The baseline Villalta Scale score is usually identified as an independent predictor (9). Proximal, recurrent ipsilateral, or provoked DVT; previous varicose vein surgery; body mass index (BMI); age; gender; smoking status; and persistent venous obstruction may be helpful for risk stratification (10, 11). Five prediction models were developed by Huang et al. (12), the two-step model by Amin et al. (13), the SOX-PTS score by Rabinovich et al. (14), the prediction model for the elderly by Méan et al. (15), and a new predictive model by Qiu et al. (16) to determine the probability of PTS more accurately and facilitate clinical decision-making, and the two were validated externally (13, 14). An accurate clinical prognostic model can help patients at high risk of developing PTS receive sufficient clinical education and achieve optimal anticoagulation quality to prevent severe PTS and lower the cost of treatment. However, these models have limitations. First, the SOX-PTS score and two-step model were developed based on a large cohort (762 former, 479 latter); however, they had poor discrimination (13, 14). The SOX-PTS scale yielded C-Statistics of 0.65 (95% CI:0.64–0.67) and 0.63 (95% CI:0.59–0.67) in internal and external validation, respectively (14). For the two-step model, the optimism-corrected) AUCs were 0.71 for the baseline model and 0.60 for the secondary model, and those in the derivation cohort were 0.66 (95% CI:0.63–0.70) and 0.64 (95% CI:0.60–0.69), respectively, in external validation (13). Second, the other three prediction models, including the APTSD score, prediction model for the elderly, and new PTS predictive model by Qiu et al., showed far better prediction ability (AUC varied from 0.71 to 0.79); however, they were developed based on smaller cohorts (107 for APTSD score, 276 for being elderly, and 210 for the new PTS predictive model by Qiu et al.), and all lacked external validation, which made their models less convincing (12, 15, 16). Third, the model developed by Méan et al. was specially built for elderly patients aged >65 years, which undermined the applicability of the model (15). Fourth, these five models were built using traditional Cox or logistic regression (LR). However, some high-dimensional or non-linear relationships between clinical data and outcomes could not be identified.

Machine learning (ML) is a widely accepted computational technique that can overcome some of the limitations of current analytical approaches and capture high-dimensional, non-linear relationships among clinical features to make data-driven outcome predictions (17). ML can also improve the robustness and generalizability of the prediction model by constructing a phenotypically cohort-based risk model (18). The potential to improve prediction accuracy for cardiovascular diseases using ML approaches has been investigated widely (19, 20). In this study, we hypothesized that ML could help improve the prediction accuracy of PTS using numerous multidimensional clinical variables. The clinical data used for model building were obtained from the Acute Venous Thrombosis: Thrombus Removal with Adjunctive Catheter-Directed Thrombolysis (ATTRACT) study, a phase III, multi-center, dual-arm randomized clinical trial (21). The model was validated in a Chinese cohort to investigate its generalizability.

## Materials and methods

### Patients and materials

Clinical data of the derivation cohort included in this study were extracted from the ATTRACT study (21). A total of 691 patients with symptomatic proximal DVT, involving the femoral, common femoral, or iliac veins (with or without other involved ipsilateral veins), were randomly assigned to receive either pharmacomechanical catheter-directed thrombolysis (PCDT) with standard anticoagulation therapy or separate standard anticoagulation therapy in a 1:1 ratio. Relevant participant inclusion criteria can be found in the original study. Subjects were enrolled at 30–60 United States Clinical Centers for 4.5 years and followed up for 24 months. Since not every individual in ATTRACT completed a 2-year follow-up, patients with <2 years of follow-up and who did not present with PTS were excluded from this study to reduce the follow-up bias.

Clinical data in the external validation cohort were obtained from the electronic record database of Sun Yat-sen Memorial Hospital. The inclusion criteria were as follows: patients diagnosed with lower-extremity DVT who were admitted to the hospital between 2010 and 2020, and the gold standard for diagnosis was thrombus filling defect detected by Doppler ultrasound in the deep iliofemoral or femoral popliteal veins. Other auxiliary diagnoses included clinical symptoms, D-dimer index, and relative clinical score. As this study was retrospective, the requirement for informed consent was waived under the ethical supervision of the center. The patients were followed up for 2 years, and their Villalta and venous clinical severity scores (VCSS) were calculated. The exclusion criteria were as follows: (1) patients who refused follow-up visits or forgot about their status; (2) patients who had not been followed up for 2 years and did not present with PTS events; (3) patients whose baseline data could not be found in the electronic record database; (4) The patient was diagnosed with DVT but also with small saphenous vein thrombosis, femoral-popliteal vein sclerosis, and others diagnosed using Doppler ultrasonography; and (5) Patients mainly treated with traditional Chinese medicine.

### Clinical treatment

The treatment plan in the ATTRACT study can be obtained from the original study or BIOLINCC in detail. The treatment plan of the external validation cohort also included patients undergoing standard DVT treatment with or without PCDT. The basic standard DVT treatment includes anticoagulation, inferior vena cava filter implantation, and physical pressure therapy. Anticoagulation drugs consisted of unfractionated heparin, low-molecular-weight heparin (LMWH), vitamin K antagonists (VKA), and direct oral anticoagulants (DOAC). For patients with DVT, but not cancer, DOACs, such as rivaroxaban or LMWH with VKA, were preferred. For those with cancer, LMWH, as well as VKA or DOAC, was preferred. The use of retrievable inferior vena cava filters is generally recommended for patients at a high risk of pulmonary embolism (PE) (history of PE, planned use of pneumatic compression therapy). Physical pressure therapy includes the use of elastic stockings and intermittent pneumatic compression devices. The treatment plan for PCDT at our center was detailed in our previous study (22). The patients were treated with LMWH twice daily before and after PCDT. The urokinase dosage was adjusted according to the patient’s weight. Urokinase was first injected at a bolus dose of 2–3 × 10^5^ U. Urokinase was continuously infused at a dose of 1–1.5 × 10^4^ U/kg/d. Residual thrombi were evaluated daily using ultrasonography or venography. Thrombolysis should generally last for less than 7 days. If the patient experienced a mild, controllable bleeding event, PCDT was paused. If minor bleeding continued, the PCDT was permanently disabled. When anticoagulation was administered, an appropriate antagonist was used, if necessary. The center followed the guidelines of the American College of Chest Physicians for the diagnosis and treatment of DVT (23).

### Outcomes and variables definition

In 2009, the International Society on Thrombosis and Hemostasis (ISTH) recommended the Villalta scale for PTS assessment 3–6 months following acute DVT (24). The Villalta score was calculated using five subjective symptoms (pain, spasm, heaviness, itching, and paresthesia) and six clinical signs (edema, redness, induration of the skin, hyperpigmentation, venous distension, and calf compression pain) scored on a scale from 0 (non-existent) to 3 (severe). The main outcome was PTS (binary outcome, which was defined as Villalta score of ≥5), whereas moderate-severe PTS (binary outcome, which was defined as Villalta score of ≥10) and severe PTS (binary outcome, which was defined as Villalta score of ≥15) were secondary outcomes.

The VCSS score was also calculated for each patient, if possible. The VCSS score was calculated using 10 items, including pain, varicose veins, venous edema, skin pigmentation, inflammation, induration of active ulcers, number of active ulcers, active ulcer diameter, ulcer duration, and compression therapy, and scored on a scale from 0 (non-existent) to 3 (severe). The SOX-PTS score was also calculated based on the research by Rabinovich et al., which contained three items: iliofemoral DVT (1 score), BMI of ≥35 (2 scores), baseline Villalta score of ≥15 (2 scores), or baseline Villita score of 10–14 (1 score) (14).

Baseline data of the external validation cohort. Age, sex, complications, history of venous thromboembolism (VTE), provoked DVT, and in-hospital diagnoses were obtained from the admission records. The clinical treatment plan was obtained from the doctor’s list. Height and weight were obtained from the nursing sheets. The DVT type and leg involved were obtained from the Doppler ultrasonography reports.

### Imputation of missing value

Only variables that had missing value rate lower than 5% would be included in the model and filled with imputation. The missing rate of all variables is shown in Supplementary Table 1. Given the heterogeneity of the different populations in the derivation and external validation cohorts, imputation was conducted separately in two independent datasets. In this study, a single imputation was conducted to fill in the missing values based on the complete conditional criterion. Missing values were filled using the predictive mean matching method. Each missing variable was estimated using an independent model to ensure its validity (25). To ensure the authenticity of these scores, the Villalta, VCSS, and SOX-PTS scores were not imputed for missing values.

### Feature selection and model development

Twenty-three variables were included in the structured dataset: basic demographic information, including age, sex, height, weight, and BMI; DVT-associated variables, including an extension to the iliac vein or isolated femoropopliteal, DVT leg, previous VTE, major surgery, hospitalization, plaster cast immobilization, childbirth, impatient qualifying DVT, baseline Villalta score, and complications, including hypertension, diabetes mellitus, high cholesterol, asthma, chronic obstructive pulmonary disease (COPD), angina or myocardial infarction (MI), congestive heart failure (CHF), DVT treatment type, and aspirin use. The treatment included PCDT with anticoagulation or base anticoagulation only.

As there still exited imbalance in the derivation set (slight for PTS in 24 months: 327 [58.9%], mainly when the outcome was set as moderate-severe PTS in 24 months: 144 [25.9%] and severe PTS in 24 months: 69 [12.4%]), the synthetic minority over-sampling technique (SMOTE) was used to oversample the derivation set, which was intended to synthesize new samples and add them into the derivation cohort to ensure equality between the number of positive and negative examples. Our previous experimental results also had shown that the performance index of the models was improved after oversampling in both primary and secondary outcomes (Supplementary Table 2).

To decrease the effect of non-normality on the model performance, the Shapiro-Wilk normality test was conducted to detect the normality of the continued variables in the derivation cohort (including age, height, weight, BMI, and base Villalta score) and none of them showed normality (Supplementary Table 3). In the derivation and external validation cohorts, zero-mean normalization of non-normal distribution continued variables was performed to eliminate dimensionality effects and improve comparability among variables.

To select a more suitable model that had a better matching degree with the data, 12 algorithms [including random forest (RF), logistic regression (LR), gradient boosting decision tree (GBDT), extreme gradient boosting (XGB), k-Nearest Neighbors (KNN), iterative dichotomiser 3 (ID3), classification and regression trees (CART), adaptive boosting (ADB), Gaussian naive Bayes (GNB), least absolute shrinkage and selection operator (LASSO), Elasticnet, and support vector classification (SVC)] were conducted to build models, and, finally, models with better performances in both derivation and external validation cohorts, which did not have too much overfitting, were chosen (Supplementary Table 4). At last, four ML algorithms, XGBoost, GBDT, and LR based on gradient descent, L1 regularization, and RF, were used for model building. An overview of ML algorithm principles used in this study is shown in Supplementary Methods. A grid search method was used to optimize the hyperparameters to improve the prediction ability of the model. Every individual in the derivation and external validation cohorts was given a prediction probability according to the different ML models.

In addition to the primary outcome, models for predicting secondary outcomes were established and validated.

### Feature importance

The relative importance of each feature in the four models was calculated and ranked to select the predictor with the greatest impact on each outcome. The feature importance was retrieved using the scikit-learn library and XGBoost package. Based on the different principle of the four model algorithms, we used different methods to calculate the feature importance. For RF, we used feature_importances_ property of RandomForestClassifier to calculate the feature importances, and the calculation method was based on impurity. For GBDT, we used feature_importances_ property of GradientBoostingClassifier, and the calculation method was based on impurity. For XGBoost, we used feature_importances_ property of XGBClassifier, and the calculation method was based on “gain,” which used the average gain across all splits of the feature. For LR, we used coef_ attribute of LogisticRegression, and the calculation method was based on regression coefficient.

Moreover, to explain the interpretability of ML in more depth, permutation importance was calculated by ELI5 package of Python. Partial dependence plots (PDP) were drawn by the sklearn package of Python to show the marginal effect that each feature had on the predicted outcome of a model. Shapley additive explanations (SHAP) values were calculated by the SHAP package of Python, which used a game theoretic approach, to explain the output of ML models. The SHAP force plots and feature importance plots were also plotted.

### Evaluation and validation of the model

Evaluation of the model was internally validated with 10-fold cross-validation in the derivation cohort to investigate the stability of the model (derivation cohort was divided into training and internal validation dataset for 10-fold cross-validation), and then external validation was conducted to investigate the generalization ability of the model. AUC and calibration plots were used to evaluate the discrimination and calibration. After determining the cutoff value of the prediction probability by the receiver operating characteristic (ROC) curve, calibration and risk classification results, F scores, negative predictive value, positive predictive value, sensitivity, and specificity were used to evaluate the risk stratification ability of the models. We also externally validated the SOX-PTS score in the derivation and external validation cohorts and used net reclassification improvement (NRI) and integrated discrimination improvement (IDI) to investigate the prediction ability improvement of ML models compared with SOX-PTS.

### Risk classification

Patients in the external validation cohort were divided into estimated risk deciles in accordance with the prediction probability yielded by the four models and then grouped into low-, intermediate-, and high-risk groups with thresholds reflecting clinically meaningful gradients in risk from one group to the next. The mean prediction probability and observed probability were calculated for each group.

### Statistical analysis

Continuous variables were represented by the median with interquartile range (IQR) and compared using the Kruskal–Wallis test. Categorical variables are expressed as percentages, compared with chi-square tests. A two-sided *P* < 0.05 was considered statistically significant. Data imputation and significance tests were conducted using R software (version 3.6.3). Data preprocessing, model development, and further evaluation and validation were conducted using Python (version 3.8.5).

### Role of funders

The funders of this research had no role in the study design, management, provision of study materials, data collection, data analysis, interpretation of the data, manuscript writing, preparation, review, and approval of this manuscript, or the decision to submit the manuscript for publication.

## Results

**FIGURE 1:**
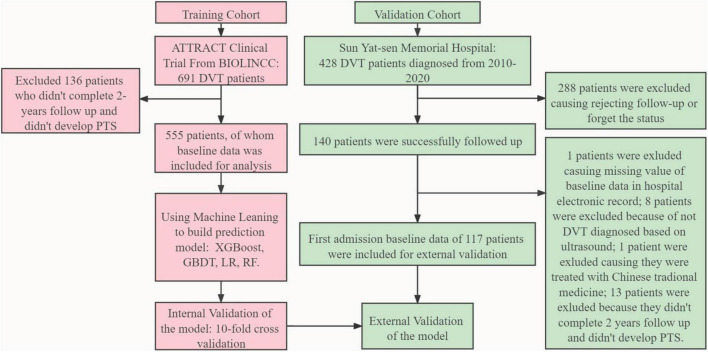
Model development and evaluation pipeline. ATTRACT, Acute Venous Thrombosis: Thrombus Removal with Adjunctive Catheter-Directed Thrombolysis; BIOLINCC, Biologic Specimen and Data Repository Information Coordinating Center; PTS, post-thrombotic syndrome; XGBoost, eXtreme gradient boosting; GBDT, gradient boosting decision tree; RF, random forest; LR, logistic regression; DVT, deep vein thrombosis.

After filtration based on the exclusion criteria in this study, 555 patients from the ATTRACT study were finally included in the derivation cohort to build four models with ML methods comprising XGBoost, GBDT, LR, and RF. As for the external validation cohort, 428 patients were diagnosed with DVT between 2010 and 2020, 288 patients refused follow-up or forgot the details of body status, and 117 patients were finally included in the external validation cohort. The study pipeline is illustrated in Figure 1. The baseline data of the derivation and external validation cohorts are shown in Table 1, and the heterogeneity of different populations can be observed. The prevalence of PTS occurrence within 2 years and previous VTE was higher in the derivation cohort than in the external validation cohort (58.9 vs. 32.5% [*P* < 0.001]; 23.4 vs. 19.7% [*P* = 0.446]). The BMI in the derivation cohort was higher than that in the validation cohort (30.84 [26.98, 36.17] vs. 23.87 [21.31, 26.20], *P* < 0.001), as were basic comorbidities and aspirin use (21.4 vs. 10.3%, [*P* = 0.008]). However, the DVT occurrence age was lower in the derivation cohort than in the external validation cohort (54 [44, 62] vs. 59 [48, 67], *P* = 0.002). A higher prevalence of VTE occurrence, and basic comorbidities, as well as a higher BMI, might be associated with a higher prevalence of PTS occurrence.

**TABLE 1:** Baseline characteristics and outcome of derivation cohort and validation cohort.

Characteristics and outcome	Derivation cohort (*n* = 555)	Validation cohort (*n* = 117)	*P-*value
Treatment			0.011
Using PCDT with anticoagulation	279 (50.3%)	43 (36.8%)	
Using anticoagulation only	276 (49.7%)	74 (63.2%)	
DVT type			0.055
Extend to Iliac vein	313 (56.4%)	54 (46.2%)	
Isolated femoropopliteal	242 (43.6%)	63 (53.8%)	
Age	54.00 [44.00, 62.00]	59.00 [48.00, 67.00]	0.002
Gender			0.004
Male	349 (62.9%)	56 (47.9%)	
Female	206 (37.1%)	61 (52.1%)	
**Comorbidity**			
Hypertension	242 (43.6%)	15 (12.8%)	<0.001
Diabetes mellitus	91 (16.4%)	8 (6.8%)	0.012
High cholesterol	176 (31.7%)	8 (6.8%)	<0.001
Asthma	57 (10.3%)	3 (2.6%)	0.013
COPD	22 (4.0%)	2 (1.7%)	0.357
MI	25 (4.5%)	4 (3.4%)	0.783
CHF	26 (4.7%)	1 (0.9%)	0.097
Height	175.00 [165.10, 182.88]	164.00 [156.00, 170.00]	<0.001
Weight	93.00 [80.95, 112.14]	62.50 [57.00, 71.50]	<0.001
BMI	30.84 [26.98, 36.17]	23.87 [21.31, 26.20]	<0.001
**DVT leg**			0.029
Right	209 (37.7%)	31 (26.5%)	
Left	346 (62.3%)	86 (73.5%)	
Previous VTE	130 (23.4%)	23 (19.7%)	0.446
**DVT risk factor**			
Major surgery	48 (8.6%)	25 (21.4%)	<0.001
Hospitalization	55 (9.9%)	14 (12.0%)	0.618
Plaster cast immob	15 (2.7%)	3 (2.6%)	1
Childbirth	7 (1.3%)	5 (4.3%)	0.064
Inpatient qualify DVT	92 (16.6%)	17 (14.5%)	0.683
Taken aspirin	119 (21.4%)	12 (10.3%)	0.008
SOX-PTS score	2.00 [1.00, 3.00]	1.00 [0.00, 1.00]	<0.001
PTS in 24 Months	327 (58.9%)	38 (32.5%)	<0.001

PCDT, pharmacomechanical catheter-directed thrombolysis; DVT, deep vein thrombosis; COPD, chronic obstructive pulmonary disease; MI, myocardial infarction; CHF, congestive heart failure; BMI, body mass index; VTE, venous thromboembolism; PTS, post-thrombotic syndrome.

All values included in the machine learning model had missing value rate lower than 1%.

**FIGURE 2:**
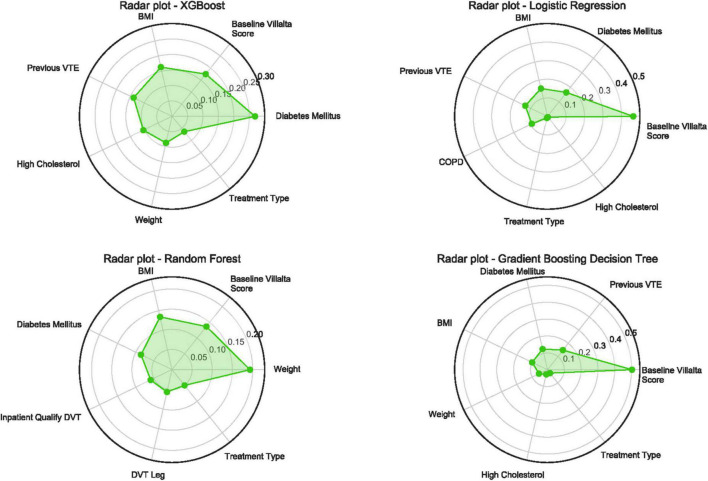
Radar plot for the seven important predictors of post-thrombotic syndrome in 24 months. Higher value means more importance of the features determined by different ML algorithms. PTS, post-thrombotic syndrome; XGBoost, eXtreme gradient boosting; VTE, venous thromboembolism; BMI, body mass index; COPD, chronic obstructive pulmonary disease; DVT, deep vein thrombosis; ML, machine learning.

The relative importance of the features in the four models was ranked, and a radar plot of the seven most important features for each model is shown in Figure 2. The main predictors varied among the four models, which was due to the principle of different algorithms and the method of importance calculation. BMI, diabetes mellitus, baseline Villalta score, and treatment type all appeared on the importance radar of the four models. High-cholesterol level, weight, and history of VTE were observed on radar images in three of the models. In addition, two other radar plots built for predicting the occurrence of moderate-severe PTS and severe PTS are shown in Supplementary Figures 1, 2. It is worth noting that BMI, diabetes mellitus, baseline Villalta score, and treatment type were four significant features that appeared in all radar plots, whereas BMI failed to appear in the GBDT model when predicting moderate-to-severe PTS. Thus, there were adequate reasons to believe that these four features were the most important for predicting PTS.

To explain the interpretability of ML in more depth and evaluate the effect of variables on outcome, permutation importance for 3 outcomes was calculated and is shown in Supplementary Tables 5–7. Baseline Villalta score got the highest weight in LR and GBDT when predicting PTS in 24 months, while weight and diabetes mellitus got highest in RF and XGB. PDP showed the influence of each feature for 3 outcomes and is shown in Supplementary Figures 3–5. The SHAP force plots showed which features have the most influence on the model’s prediction for a single observation and are shown in Supplementary Figures 6–8. The SHAP feature importance plots are shown in Supplementary Figures 9–11, which were similar to permutation importance, showing the effect of each feature on the outcome.

**FIGURE 3:**
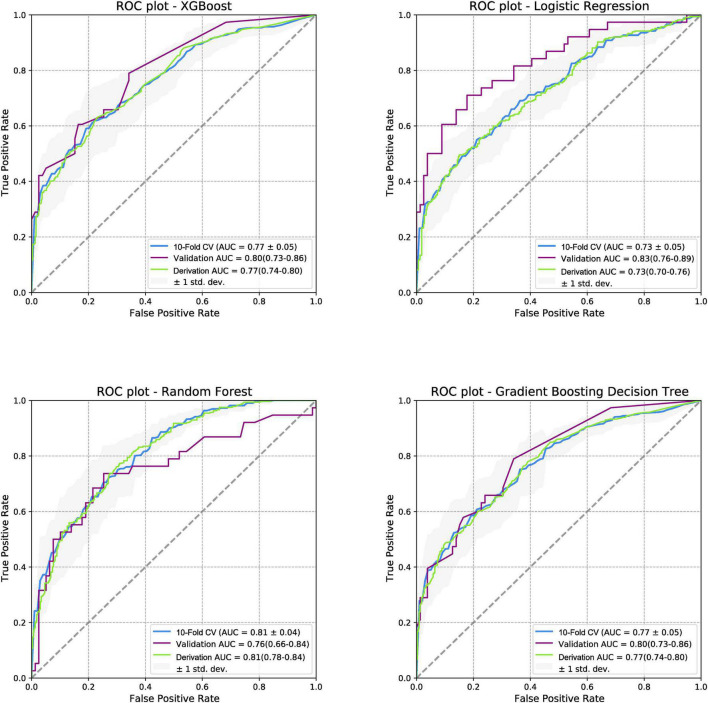
Receiver operating characteristic curves for post-thrombotic syndrome at 2-year follow-up. ROC, receiver operating characteristic curve; PTS, post-thrombotic syndrome; AUC, area under the curve; XGboost, eXtreme gradient boosting.

When the models were evaluated and validated, the LR, based on gradient descent and L1 regularization, performed best in external validation (0.83 [95% CI:0.76–0.89]). In the derivation cohort, RF performed best (0.81 [95% CI:0.78–0.84]), whereas LR performed worst (0.73 [95% CI:0.70–0.76]). The ROC curves for the four models for predicting PTS are shown in Figure 3. In addition, four models were used to predict moderate-to-severe PTS and severe PTS. In the external validation cohort, LR performed best in predicting moderate-to-severe PTS and PTS, with AUCs of 0.97 (95% CI:0.94–1) and 0.99 (95% CI:0.97–1), respectively. The ROC curves for the prediction of secondary outcomes are shown in Supplementary Figures 12, 13.

The calibration plots of the four models for the three outcomes in both the derivation and external validation cohorts were also plotted, as shown in Supplementary Figures 14–19.

For other ML performance indices, we paid more attention to the F2 score because the dataset had a degree of imbalance, and the potential cost of missed real cases was higher than that of missed cases. The results showed that all four models had a good predictive ability (F2 score:0.70–0.76 when the threshold was set to 0.3). LR performed best in predicting moderate-to-severe and severe PTS (F2 score:0.76 and 0.91, respectively, when the threshold was set at 0.4). Other performance indices (F1 score, F.5, negative predictive value, positive predictive value, accuracy, sensitivity, and specificity) are shown in Supplementary Figures 20, 21. The NRI and IDI results showed that all four models performed better than the SOX-PTS score in the derivation and external validation cohorts (NRI and IDI > 0, *P* < 0.05), as shown in Table 2.

**TABLE 2:** Net reclassification improvement and integrated discrimination improvement results of machine learning models compared with the SOX-PTS score.

Different methods compared with SOX-PTS	Derivation cohort		Validation cohort	
	NRI (95% CI/*P*-value)	IDI (95% CI/*P*-value)	NRI (95% CI/*P*-value)	IDI (95% CI/*P*-value)
XGBoost	0.621 (0.461–0.782/<0.001)	0.098 (0.074–0.121/<0.001)	0.351 (0.095–0.607/0.007)	0.176 (0.091– 0.260/<0.001)
LR	0.642 (0.484–0.801/<0.001)	0.082 (0.062–0.103/<0.001)	0.518 (0.264–0.772/<0.001)	0.239 (0.154–0.324/<0.001)
RF	0.664 (0.507–0.820/<0.001)	0.124 (0.099–0.149/<0.001)	0.350 (0.077–0.622/0.012)	0.078 (−0.001–0.157/0.054)
GBDT	0.672 (0.514–0.830/<0.001)	0.102 (0.078–0.125/<0.001)	0.404 (0.141–0.668/0.003)	0.144 (0.062–0.227/<0.001)

XGBoost, extreme gradient boosting; LR, logistic regression; RF, random forest; GBDT, gradient boosting decision tree; NRI, net reclassification improvement; IDI, integrated discrimination improvement.

**FIGURE 4:**
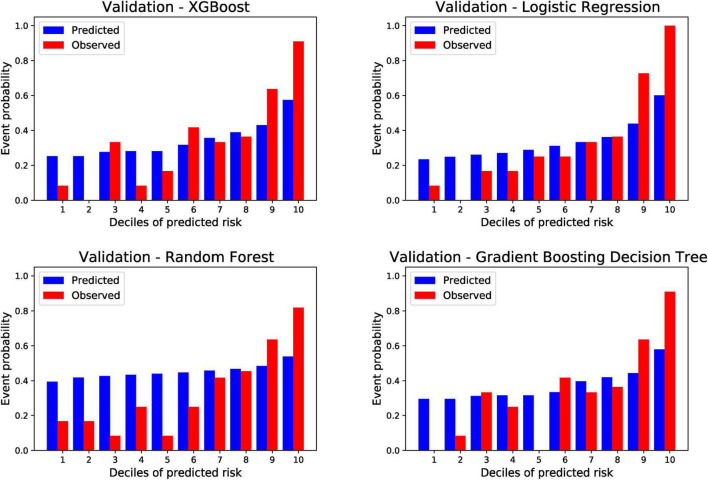
Risk of post-thrombotic syndrome within 24 months according to deciles of event probability based on four machine learning models in the validation cohort. PTS, post-thrombotic syndrome; ML, machine learning; XGBoost, eXtreme gradient boosting.

The ten divided groups based on the estimated risk deciles of the four models predicting PTS in the external validation cohort are shown in Figure 4. The observed probability also tended to increase with an increase in the prediction probability. According to the risk classification results of XGBoost, LR, and GBDT (RF was excluded because of its poor performance in external validation), we stratified the patients into three risk groups (first to fifth deciles as low risk: prediction probability approximately lower than 30%; sixth to eighth deciles as intermediate risk: prediction probability approximately of 30–40%; and eighth to tenth deciles as high risk: prediction probability approximately higher than 40%). In calibration plots of four models in both derivation and validation cohorts (Supplementary Figure 14), underestimation is shown when predicted probability is higher than 0.4. As a result, defining 0.4 as the high-risk threshold was meaningful. The risk stratification figures for PTS in the derivation cohort are also plotted and shown in Supplementary Figure 22. The risk stratification figures for moderate-to-severe and severe PTS in both the derivation and external validation cohorts are also plotted and are shown in Supplementary Figures 23–26.

To evaluate the risk classification ability of the ML models, the XGBoost model was chosen as an example to investigate the association between risk groups and clinical features. As the derivation group was divided into low-risk, intermediate-risk, and high-risk groups based on the prediction probability of XGBoost, the prevalence of PTS increased (PTS: 14.8% [9/61] low risk vs. 34.3% [12/35] intermediate risk vs. 81% [17/21] high risk; moderate-severe PTS: 0% [0/61] low risk vs. 5.7% [2/35] intermediate risk vs. 28.6% [6/21] high risk; severe PTS:0% [0/61] low risk vs. 0% [0/35] intermediate risk vs. 19% [4/21] high risk). In addition, the related risk scores at different time points showed similar results, as shown in Table 3.

**TABLE 3:** Outcome in each risk groups defined by prediction probability of the XGBoost model in external validation cohort.

Outcome	Low-risk group (*n* = 61)	Intermediate-risk group (*n* = 35)	High-risk group (*n* = 21)	*P*-value
**Risk score**				
Baseline Villalta score	1.00 [1.00, 3.00]	2.00 [1.00, 3.50]	9.00 [3.00, 13.00]	<0.001
6 Month Villalta score	0.00 [0.00, 2.00]	1.00 [0.00, 2.25]	5.00 [4.00, 10.75]	<0.001
12 Month Villalta score	0.00 [0.00, 1.00]	0.00 [0.00, 1.50]	4.00 [1.00, 6.00]	0.002
18 Month Villalta score	1.00 [0.00, 1.00]	1.00 [0.00, 2.75]	3.00 [2.00, 6.50]	0.001
24 Month Villalta score	1.00 [0.00, 2.00]	1.00 [0.00, 3.00]	3.00 [0.00, 5.25]	0.073
6 Month VCSS score	0.00 [0.00, 1.00]	1.00 [0.00, 1.25]	3.00 [2.00, 5.00]	<0.001
12 Month VCSS score	0.00 [0.00, 1.00]	0.00 [0.00, 1.00]	2.00 [1.00, 3.50]	0.002
18 Month VCSS score	1.00 [0.00, 1.00]	1.00 [0.00, 1.25]	2.00 [1.00, 3.00]	0.004
24 Month VCSS score	1.00 [0.00, 1.00]	1.00 [0.00, 2.00]	2.00 [0.00, 3.25]	0.057
Baseline SOX-PTS score	0.00 [0.00, 1.00]	0.00 [0.00, 1.00]	1.00 [1.00, 2.00]	<0.001
**Binary outcome**				
PTS in 24 months	9 (14.8%)	12 (34.3%)	17 (81.0%)	<0.001
Moderate to severe PTS in 24 months	0 (0.0%)	2 (5.7%)	6 (28.6%)	<0.001
Severe PTS in 24 months	0 (0.0%)	0 (0.0%)	4 (19.0%)	<0.001

Low-risk group was defined as patients whose XGBoost prediction ability is lower than 30%, Intermediate-risk group was defined as patients whose XGBoost prediction ability is between 30 and 40%, High-risk group was defined as patients whose XGBoost prediction ability is higher than 40%.

VCSS, venous clinical severity scores; PTS, post-thrombotic syndrome.

## Discussion

In this study, 555 patients with DVT were included to build models with different ML algorithms, and the models were validated in a Chinese cohort of 117 patients. The results showed that the models presented good prediction abilities for both the primary and secondary outcomes. When predicting PTS 2 years after acute DVT, LR based on gradient descent and L1 regularization had the highest AUC of 0.83 (95% CI:0.76–0.89) in external validation, whereas RF had the highest AUC of 0.81 (95% CI:0.78–0.84) in the derivation cohort. However, when considering the model performance in both the derivation and validation cohorts, the XGBoost and GBDT models had similar results and presented better stability and generalization. Compared with the SOX-PTS score, all ML models exhibited improved prediction ability, with NRI and IDI indices all significantly higher than zero. After dividing the external validation cohort into ten groups based on the estimated risk deciles of the models, three risk groups were identified. Moreover, a tendency of increase in the risk score and prevalence of PTS occurrence could be found as the risk increased, which indicated good clinical application of the models. Moreover, BMI, diabetes mellitus, baseline Villalta score, and treatment type were identified as important features using ML algorithms in this study. To the best of our knowledge, this study is the first to build models for predicting PTS using ML algorithms and confirm that ML can help improve the prediction ability.

Predicting and preventing PTS remains a challenge to date. There is still no effective treatment, and the management thereof relies more on prevention after DVT (8). Anticoagulation remains the cornerstone of acute DVT treatment. Although it is not the main purpose of treatment, it plays a key role in preventing the development of PTS (26). Physiological and clinical studies have shown that LMWH is preferred over VKA in preventing PTS due to its improved rates of venous recanalization and anti-inflammatory effects (27, 28). However, current guidelines do not recommend specific anticoagulation to prevent PTS in clinical practice (10). Extending anticoagulation therapy also adversely affected the prevention of PTS, and it is recommended that VTE occurrence besides PTS be prevented (29, 30). Early thrombus removal may prevent PTS by reconstructing the microenvironment of blood circulation and preserving venous function (31). However, the results of the ATTRACT study showed that PCDT did not exert a protective effect on PTS (46.7% PCDT vs. 48.2% no PCDT, *P* = 0.56). This might be interpreted as the ATTRACT study, including both femoropopliteal and iliofemoral DVT, and femoropopliteal DVT showed a lower risk of developing PTS (21). Therefore, another study conducted a subgroup analysis of the iliofemoral arm in ATTRACT and showed that PCDT reduced the risk of moderate-to-severe PTS (18% PCDT vs. 28% no PCDT, *P* = 0.021) (32). A recent study investigated the efficacy of different treatment modalities for percutaneous thrombus removal and found that the use of PCDT for treating iliofemoral DVT could provide comparable patient outcomes, comparable vessel patency, an acceptable safety profile, and a reduced overall lytic dose (33). Percutaneous mechanical thrombectomy (PMT) is an alternative method for DVT treatment, and pharmacomechanical thrombectomy refers to a combination of mechanical and pharmacological therapies to achieve thrombolysis. Compared with PCDT or catheter-directed thrombolysis (CDT) alone, pharmacomechanical thrombectomy can lower thrombolytic dosage and procedural time and achieve a more complete resolution of the thrombus. When the prognosis results of PMT ± CDT and CDT alone were compared, the partial thrombolysis rate was higher in the PMT ± CDT group (odds ratio, 2.64; 95% confidence interval, 1.34–5.21; *P* = 0.005) (34). With advancements in endovascular technology, we believe that it can reduce PTS risk in the future. An accurate prediction model can help identify patients who can benefit from endovascular surgery, which we hypothesize is one of the most important implications of our models.

Machine learning is currently an effective method to investigate high-dimensional and non-linear relations between features and outcomes and improves the prediction ability of the prognostic model (17). In this study, ML models reached a higher AUC than previous PTS models and attained an improved prediction ability compared with SOX-PTS. We used an American cohort to build models and a Chinese cohort to validate them to ascertain whether the models were effective in other populations as well. Western and Asian populations are extremely heterogeneous. Previous studies have indicated that VTE occurrence and reoccurrence were not as high in the Chinese cohort as in the Western population (35, 36). In addition to the effect of genes, nutritional status, dietary habits, economic status, and medical status also affect the prognosis of DVT. In this study, we found that the prevalence of PTS occurrence in 2 years and previous VTE was higher in the derivation cohort, which might be associated with higher BMI, higher prevalence of basic comorbidity, and aspirin use (aspirin use also indicated worse health status). However, models built with XGBoost and GBDT still showed good and stable prediction abilities in internal and external validation, which indicated the good stability and generalization of ML.

The chosen four ML models in this study both had their advantages and disadvantages. LR is a kind of discriminative models, which can be used in combination with regularization methods. The linear models have high interpretability compared to most classification algorithms. LR has the advantages of easy implementation and low computational cost. However, when there are a large number of features, LR performances are poor, and LR is easy to cause underfitting. It is mostly used to deal with binary classification problems, and the classes must be linearly separable. The non-linear characteristics need to be transformed before modeling. RF performs well on a lot of datasets. Firs, RF is suitable for highly dimensional features. Second, it has fast calculation speed and easy implementation. Third, the model has strong generalization ability. Fourth, RF has strong anti-interference capability and can also be used when there is a large amount of missing data. Fifth, it has a strong anti-overfitting ability. The disadvantages of RF are the following: poor performances in solving regression problems; the model is similar to the black box, which has poor interpretability; and it may not produce good classification results in small samples or low dimensional data. GBDT has good prediction performances and is suitable for low-dimensional data. It can flexibly handle various types of data and has strong robustness to outliers. However, due to the dependency between weak learners, it is difficult to carry out parallel computing. The calculation complexity will be increased when the data dimension is high. XGBoost is based on GBDT. Compared with GBDT, XGBoost has the following advantages: first, adding the complexity of tree models to the regularization term, the generalization ability is better; second, it uses Taylor expansion on the loss function to accelerate the optimization speed; and third, XGBoost supports parallel processing (37).

The performance metrics for four ML models in predicting different outcomes were shown in Supplementary Figure 21. The threshold term indicated the threshold of 0/1 classification of samples according to the model prediction probability, where in the fourth column of each model was the best cutoff value of ROC curve. For PTS in 24 months, performance indices of RF and LR were similar, performance indices of GBDT and XGBoost are similar, and the former two models performed better than the latter two. For moderate-severe PTS, performance indices of four models were different. LR had the highest F2 score and accuracy, while the other three models had low F2 scores and accuracy. XGBoost had the highest sensitivity and specificity, while LR had the lowest sensitivity and specificity. For severe PTS, LR had the best performance indices, while XGBoost and GBDT had the worst performance indices, because both the accuracy and sensitivity of LR were higher than XGBoost and GBDT.

Area under the curve is an important index to evaluate the discrimination of the models; however, the cut-off value of prediction probability should also be focused on because risk stratification and prevalence identification in each risk group are important for clinical decision-making. Based on the prediction results of the XGboost, three risk groups were identified. PTS occurrence reached up to 81% in the high-risk group and only 14.8% in the low-risk group. Other ML performance indices can also be calculated after the threshold is determined to reflect the effectiveness of the models. The results showed that all four models had good predictive ability (F2 score:0.70–0.76 when the threshold was set to 0.3). LR performed best in predicting moderate-severe and severe PTS (0.76 and 0.91 when the threshold was set as 0.4). The threshold may vary in different populations as the prevalence of disease may differ.

In this study, a more important result was the identification of important features for predicting PTS, including BMI, diabetes mellitus, baseline Villalta score, treatment type, high cholesterol level, and history of VTE. BMI and baseline Villalta score as risk factors have been validated by previous studies and were also the predictive items included in the SOX-PTS score (14). A history of VTE or recurrent DVT is a strong predictor of PTS (38). PCDT was found to be a protective factor in the present study. Therefore, diabetes mellitus and high-cholesterol levels were identified as new risk factors that have not been reported elsewhere. The pathophysiology and epidemiological mechanisms are complex. Diabetes mellitus is associated with an increased risk of DVT and CVI (39, 40), which can be attributed to PTS. Moreover, a hyperglycemic environment can damage the vascular wall and create hypercoagulability (41). High-cholesterol levels are also associated with DVT due to hypercoagulability in the blood and can reduce the rate of thrombosis recanalization (42). Previous studies have shown that statin use was associated with a higher rate of thrombus resolution and could reduce the rate of PTS (38.3 and 48.5% in the statin and control groups, respectively, *P* = 0.02) (43, 44). Hyperglycemic and high-cholesterol levels can also contribute to inflammation and senescent pathological changes in the vasculature (45, 46). However, there are still some confounding factors, such as BMI, diabetes mellitus, and high-cholesterol levels, which are associated with BMI and drug use. The mechanism, by which these two factors contribute to the development of PTS, requires further investigation.

This study has several limitations. First, it had a retrospective design, and the derivation cohort in the ATTRACT study was not designed to build models in the original study. Consequently, some important factors were not included in the model because they were not included prospectively or too many values were missing. For example, some laboratory induces, such as D-dimers, were not included because >20% of the values were missing, which might affect the ability of the model if they were filled using mean values or single imputation. Moreover, previous studies have indicated that previous varicose vein surgery is a strong predictor (13). However, it was not recorded in the ATTRACT database and not included in the model. Second, as varicose veins, iliac vein compression syndrome, and smoking status were not recorded in ATTRACT, other previous scores [including APTSD score by Huang et al. (12), two-step model by Amin et al. (13), the prediction model for elderly by Méan et al. (15), and a new predictive model by Qiu et al. (16)] could not be validated and compared with our models. Although the AUC showed that our ML models improved the prediction ability, it would be more rigorous if they were validated in the same cohort using the NRI and IDI to evaluate the difference in prediction ability. Third, the external validation cohort did not record any bleeding events as a safety outcome. If bleeding events were recorded, the prevalence of PTS and bleeding events could be calculated in each risk group, e.g., using the PRAISE score (20), which could guide further anticoagulation or other treatment. However, the duration of anticoagulation therapy cannot be extended, especially to prevent PTS. In clinical practice, extended anticoagulation should refer to the risk of VTE and bleeding. We believe that this limitation did not affect the value of the model too greatly. Fourth, all four models slightly underestimated the high-risk category when predicting PTS, which negatively impacted the predictive power of the prediction system. In the future, we may consider ways to reduce the underestimation of the current model. For example, adding a penalty during the derivation phase or selecting a different classification threshold. Other ML algorithms, such as neural networks, can be used to build the model or increase the sample size of the derivation data.

In conclusion, we developed and validated models using ML algorithms in large cohorts. This study demonstrated that the ML models had accurate prediction ability and stable generalization, which can further facilitate clinical decision-making, with potentially important implications for selecting patients who will benefit from endovascular surgery.

## Data availability statement

The raw data supporting the conclusions of this article will be made available by the authors, without undue reservation.

## Ethics statement

Informed patient consent was exempted, and the study was approved by the Ethics Committee of Sun Yet-sen Memorial Hospital. Additionally, the study was conducted in accordance with the ethical principles for medical research involving human subjects set out in the Declaration of Helsinki. Data associated with ATTRACT research were requested from the Biologic Specimen and Data Repository Information Coordinating Center (BIOLINCC). According to the purposes of the study, the clinical research protocol was written in advance and reviewed by the Ethics Committee of Sun Yat-sen Memorial Hospital (SYSEC-KY-KS-2020-188) and the Independent Committee of BIOLINCC (BIOLINCC ID:9456). Related baseline data and outcomes were consolidated and extracted using BIOLINCC software.

## Author contributions

TY, RS, GY, and KH: conception and design. TY and KH: administrative support. RS, GY, LL, DZ, and JX: provision of study materials or patients. RS and LL: collection and assembly of data. RS, GY, LL, SK, XW, and ZX: data analysis and interpretation. All authors manuscript writing, final approval of manuscript, agreed to be accountable for all aspects of the work in ensuring that questions related to the accuracy or integrity of any part of the work are appropriately investigated, and resolved.

## Abbreviations

PTS: post-thrombotic syndrome
ML: machine learning
DVT: deep vein thrombosis
LR: logistic regression
AUC: area under the curve
95% CI: 95% confidence intervals
XGBoost: eXtreme gradient boosting
GBDT: gradient boosting decision tree
CVI: chronic venous insufficiency
PE: pulmonary embolism
BMI: body mass index
ATTRACT: Acute Venous Thrombosis, Thrombus Removal with Adjunctive Catheter-Directed Thrombolysis
PCDT: pharmacomechanical catheter-directed thrombolysis
BIOLINCC: Biologic specimen and Data Repository Information Coordinating Center
VCSS: venous clinical severity scores
VKA: vitamin K antagonists
LMWH: low-molecular weight heparin
DOAC: direct oral anticoagulation
ISTH: International Society on Thrombosis and Hemostasis
VTE: venous thromboembolism
COPD: chronic obstructive pulmonary disease
MI: myocardial infarction
CHF: congestive heart failure
SMOTE: Synthetic Minority Over-sampling Technique
RF: Random Forest
NRI: net reclassification improvement
IDI: integrated discrimination improvement
IQR: interquartile range
ROC: receiver operating characteristic
PMT: percutaneous mechanical thrombectomy
CDT: catheter directed thrombolysis.
