# Supplementary material for: Machine learning-based prediction of the post-thrombotic syndrome: Model development and validation study
Source: Front Cardiovasc Med. 2022 Sep 16;9:990788. doi: 10.3389/fcvm.2022.990788 (PMC9523080; doi:10.3389/fcvm.2022.990788)
Supplement: Supplementary file 1 [file Data_Sheet_1.docx]

Title: Machine learning-based prediction of post-thrombotic syndrome: a model development and validation study

**Supplementary Files**

[Supplementary Methods 3](#_Toc111636786)

[Supplementary Table 1 Missing rate of value in derivation cohort and training cohort. 4](#_Toc111636787)

[Supplementary Table 2 AUC values of the four models with and without oversampling 5](#_Toc111636788)

[Supplementary Table 3 Normality detection of the continued variables. 6](#_Toc111636789)

[Supplementary Table 4 AUC values of 12 machine learning models 7](#_Toc111636790)

[Supplementary Figure 1 Radar Plot for the seven important predictors of moderate-severe PTS in 24 months 8](#_Toc111636791)

[Supplementary Figure 2 Radar Plot for the seven important predictors of severe PTS in 24 months 9](#_Toc111636792)

[Supplementary Table 5 Permutation importance for the 23 features of PTS in 24 months 10](#_Toc111636793)

[Supplementary Table 6 Permutation importance for the 23 features of moderate-severe PTS in 24 months 11](#_Toc111636794)

[Supplementary Table 7 Permutation importance for the 23 features of severe PTS in 24 months. 12](#_Toc111636795)

[Supplementary Figure 3 PDP for the 23 features of PTS in 24 months 13](#_Toc111636796)

[Supplementary Figure 4 PDP for the 23 features of moderate-severe PTS in 24 months 17](#_Toc111636797)

[Supplementary Figure 5 PDP for the 23 features of severe PTS in 24 months 21](#_Toc111636798)

[Supplementary Figure 6 The SHAP force plot of PTS in 24 months 25](#_Toc111636799)

[Supplementary Figure 7 The SHAP force plot of moderate-severe PTS in 24 months 27](#_Toc111636800)

[Supplementary Figure 8 The SHAP force plot of severe PTS in 24 months 29](#_Toc111636801)

[Supplementary Figure 9 The feature importance plot of PTS in 24 months 31](#_Toc111636802)

[Supplementary Figure 10 The feature importance plot of moderate-severe PTS in 24 months 32](#_Toc111636803)

[Supplementary Figure 11 The feature importance plot of severe PTS in 24 months 33](#_Toc111636804)

[Supplementary Figure 12 ROC curves for moderate-severe PTS at 2-year follow-up 34](#_Toc111636805)

[Supplementary Figure 13 ROC curves for severe PTS at 2-year follow-up 35](#_Toc111636806)

[Supplementary Figure 14 Calibration curve of ML models built for PTS in external validation cohort 36](#_Toc111636807)

[Supplementary Figure 15 Calibration curve of ML models built for PTS in derivation cohort 37](#_Toc111636808)

[Supplementary Figure 16 Calibration curve of ML models built for moderate-severe PTS in external validation cohort 38](#_Toc111636809)

[Supplementary Figure 17 Calibration curve of ML models built for moderate-severe PTS in derivation cohort 39](#_Toc111636810)

[Supplementary Figure 18 Calibration curve of ML models built for severe PTS in external validation cohort 40](#_Toc111636811)

[Supplementary Figure 19 Calibration curve of ML models built for severe PTS in derivation cohort 41](#_Toc111636812)

[Supplementary Figure 20 AUC of four ML models in predicting different outcomes 42](#_Toc111636813)

[Supplementary Figure 21 Comparison of performance metrics for four ML models in predicting different outcomes 43](#_Toc111636814)

[Supplementary Figure 22 Risk of PTS in 24 months according to deciles of event probability based on four ML models in derivation cohort 44](#_Toc111636815)

[Supplementary Figure 23 Risk of moderate-severe PTS in 24 months according to deciles of event probability based on four ML models in external validation cohort 45](#_Toc111636816)

[Supplementary Figure 24 Risk of moderate-severe PTS in 24 months according to deciles of event probability based on four ML models in derivation cohort 46](#_Toc111636817)

[Supplementary Figure 25 Risk of severe PTS in 24 months according to deciles of event probability based on four ML models in external validation cohort 47](#_Toc111636818)

[Supplementary Figure 26 Risk of severe PTS in 24 months according to deciles of event probability based on four ML models in derivation cohort 48](#_Toc111636819)

# Supplementary Methods

**An overview of machine learning algorithm principles used in this study**

The logistic regression (LR) algorithm is a kind of linear regression model, which assumes that the data follows Bernoulli distribution, and is solved by maximum likelihood estimation and gradient descent method, so as to carry out binary classification. LR is supported by the linear regression theory and the sigmoid function is introduced. Therefore, the 0/1 distribution problem can be easily handled.

The random forest (RF) algorithm utilizes both bagging method and feature randomness to construct an uncorrelated forest of decision trees. The basic concept of RF classifier is: firstly, *k* samples are extracted from the original dataset by bootstrap sampling, and the sample size of each sample is the same as the original training set; then, *k* decision tree models are established for *k* samples, and *k* classification results are obtained; finally, vote on each record according to the *k* classification results to determine its final classification.

The gradient boosting decision tree (GBDT) algorithm is a kind of additive model that combines a series of weak classifiers into strong classifier. Unlike the traditional gradient algorithms, which only focus on the weights of the correct samples and the wrong samples, GBDT uses the forward distribution algorithm to increase the weights of the incorrectly classified samples and forme a new decision tree.

The eXtreme gradient boosting (XGBoost) algorithm is based on gradient boosting decision tree. XGBoost performs second-order Taylor expansion on the loss function, and obtains the optimal solution for the regularization term. It takes full advantage of the parallel computing of multicore CPUs to improve the calculation accuracy and speed.

**Interpretability in machine learning**

Some machine learning models are called “black boxes” because they are not interpretable by themselves. Therefore, the “interpretability” is a very active area of investigation. Due to interpretability, researchers can understand how models work and measure the effects of trade-offs in models. In this study, permutation importance were calculated by ELI5 package of Python and the outcomes are shown in Supplementary Tables 5-7. Permutation importance is defined as a method to measure how sensitive the prediction is to change in the values of each feature. Permutation importance is measured by calculating the decrease in the model score after permuting a feature. A feature is considered to be “important” if shuffling its values decreases the model score; a feature is considered to be “unimportant” if shuffling its values hardly changes the model score. Partial Dependence Plots (PDP) were drawn by sklearn package of Python to show the marginal effect that each feature has on the predicted outcome of a model and the outcomes are shown in Supplementary Figures 3-5. PDP can show whether the relationship between the targets and a feature is linear, monotonic or more complex. SHapley Additive exPlanations (SHAP) values were calculated by SHAP package of Python, which uses a game theoretic approach, to explain the output of machine learning models. Shapley values give a metric for evaluating the importance of a feature to other features. These values take into account how the loss function is affected by that feature. These values also indicate the direction of the relationship between the feature and the target. The SHAP force plots and feature importance plots were shown in Supplementary Figures 6-11. The mean absolute Shapley values are measured as SHAP feature importance. The SHAP force plot shows which features has the most influence on the model’s prediction for a single observation. Each vertical line in the longitudinal direction represents the feature importances of a single observation. And all observations stack horizontally to constitute force plot.

# Supplementary Table 1 Missing rate of value in derivation cohort and training cohort.

|  | Derivation cohort  (N = 555) | | External validation cohort  (N = 117) | |
| --- | --- | --- | --- | --- |
| Baseline Data | Missing Value | Missing Rate | Missing Value | Missing Rate |
| Age | 1 | 0.2% | NA | NA |
| Asthma | 1 | 0.2% | NA | NA |
| COPD | 1 | 0.2% | NA | NA |
| MI | 1 | 0.2% | NA | NA |
| CHF | 1 | 0.2% | NA | NA |
| Height | 2 | 0.4% | 18 | 15% |
| Weight | NA | NA | 13 | 11% |
| Childbirth | 1 | 0.2% | NA | NA |
| Inpatient Qualify DVT | 1 | 0.2% | NA | NA |
| Taken Aspirin | 1 | 0.2% | NA | NA |
| Outcome Data |  |  |  |  |
| 6 Month Villalta score | 28 | 5.0% | 22 | 19% |
| 12 Month Villalta score | 53 | 9.5% | 32 | 27% |
| 18 Month Villalta score | 99 | 17.8% | 47 | 40% |
| 24 Month Villalta score | 57 | 10.3% | 5 | 4% |
| 6 Month VCSS score | 35 | 6.3% | 22 | 19% |
| 12 Month VCSS score | 65 | 11.7% | 32 | 27% |
| 18 Month VCSS score | 110 | 19.8% | 47 | 40% |
| 24 Month VCSS score | 109 | 19.6% | 5 | 4% |

Abbreviations: DVT, deep vein thrombosis; COPD, chronic obstructive pulmonary disease; MI, myocardial infarction; CHF, congestive heart failure; NA, not available. NA means no missing value.

# Supplementary Table 2 AUC values of the four models with and without oversampling

|  |  | PTS | | moderate-severe PTS | | severe PTS | |
| --- | --- | --- | --- | --- | --- | --- | --- |
|  |  | no oversampling | oversampling | no oversampling | oversampling | no oversampling | oversampling |
| RF | train | 0.75 | 0.81 | 0.75 | 0.88 | 0.75 | 0.86 |
|  | test | 0.72 | 0.76 | 0.86 | 0.90 | 0.98 | 0.93 |
| LR | train | 0.71 | 0.73 | 0.73 | 0.76 | 0.78 | 0.82 |
|  | test | 0.83 | 0.83 | 0.97 | 0.97 | 1.00 | 0.99 |
| GBDT | train | 0.79 | 0.77 | 0.75 | 0.84 | 0.83 | 0.96 |
|  | test | 0.79 | 0.80 | 0.88 | 0.95 | 1.00 | 0.94 |
| XGB | train | 0.74 | 0.77 | 0.79 | 0.90 | 0.88 | 0.97 |
|  | test | 0.76 | 0.80 | 0.86 | 0.93 | 0.98 | 0.95 |

Abbreviations: AUC, area under the curve; PTS, post-thrombotic syndrome; XGB, extreme gradient boosting; LR, logistic regression; RF, random forest; GBDT, gradient boosting decision tree.

# Supplementary Table 3 Normality detection of the continued variables.

|  | Shapiro-Wilk normality test | |
| --- | --- | --- |
|  | *W* statistic | *P* value |
| Age | 0.969 | 1.97E-9 |
| Height | 0.984 | 9.33E-6 |
| Weight | 0.971 | 4.48E-9 |
| BMI | 0.954 | 4.36E-12 |
| Base Villalta score | 0.972 | 9.15E-9 |

Abbreviations: BMI, body mass index.

# Supplementary Table 4 AUC values of 12 machine learning models

|  | PTS | | moderate-severe PTS | | severe PTS | |
| --- | --- | --- | --- | --- | --- | --- |
|  | train | test | train | test | train | test |
| RF | 0.81 | 0.76 | 0.88 | 0.90 | 0.86 | 0.93 |
| LR | 0.73 | 0.83 | 0.76 | 0.97 | 0.82 | 0.99 |
| GBDT | 0.77 | 0.80 | 0.84 | 0.95 | 0.96 | 0.94 |
| XGB | 0.77 | 0.80 | 0.90 | 0.93 | 0.97 | 0.95 |
| kNN | 0.83 | 0.68 | 0.88 | 0.86 | 0.96 | 0.87 |
| ID3 | 0.72 | 0.73 | 0.78 | 0.85 | 0.89 | 0.91 |
| CART | 0.74 | 0.71 | 0.79 | 0.83 | 0.91 | 0.71 |
| ADB | 0.77 | 0.78 | 0.84 | 0.76 | 0.93 | 0.99 |
| GNB | 0.72 | 0.66 | 0.74 | 0.84 | 0.84 | 0.96 |
| LASSO | 0.73 | 0.83 | 0.74 | 0.97 | 0.79 | 1.00 |
| Elasticnet | 0.74 | 0.82 | 0.75 | 0.97 | 0.80 | 1.00 |
| SVC | 0.77 | 0.74 | 0.76 | 0.97 | 0.82 | 0.97 |

The blue part shows the four best performance models selected in this study.

Abbreviations: AUC, area under the curve; RF, random forest; LR, logistic regression; GBDT, gradient boosting decision tree; XGB, extreme gradient boosting; kNN, k-nearest neighbors; ID3, iterative dichotomiser 3; CART, classification and regression trees; ADB, adaptive boosting; GNB, gaussian naive bayes; LASSO, least absolute shrinkage and selection operator; SVC, support vector classification.

# Supplementary Figure 1 Radar Plot for the seven important predictors of moderate-severe PTS in 24 months


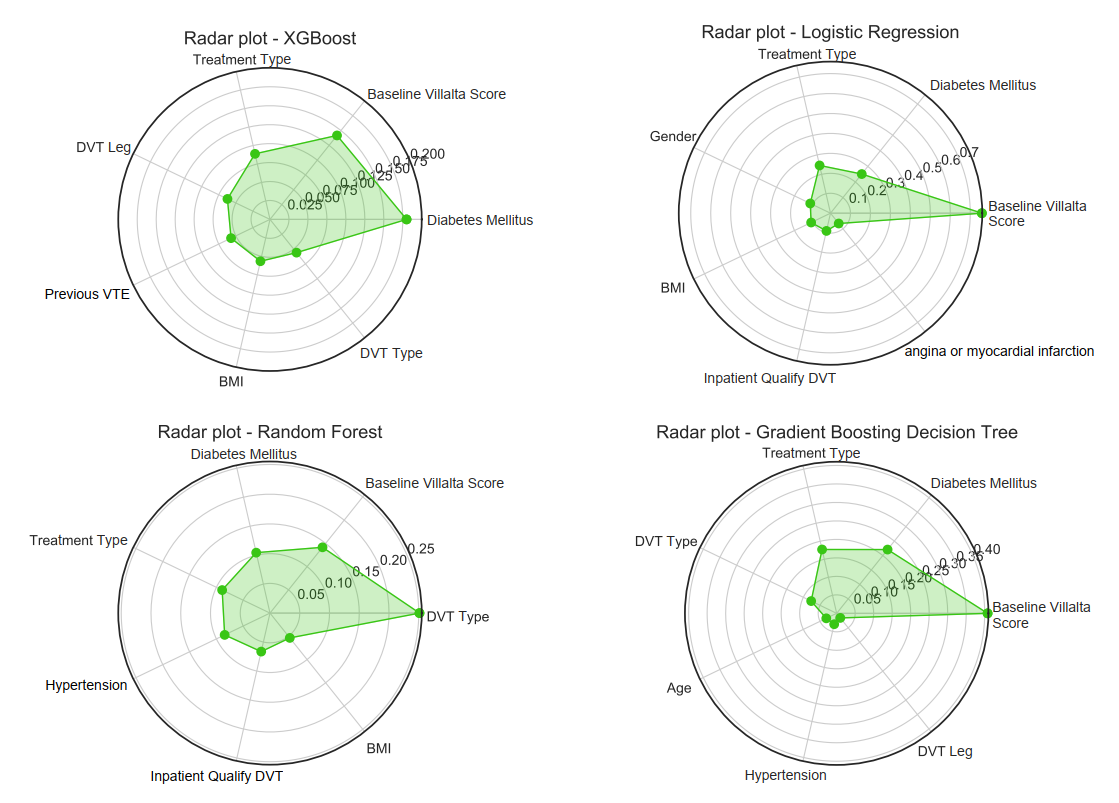


Higher value means more importance of the features determined by different ML algorithms.

Abbreviations: PTS, post-thrombotic syndrome; XGBoost, eXtreme gradient boosting; VTE, venous thromboembolism; BMI, body mass index; COPD, chronic obstructive pulmonary disease; DVT, deep vein thrombosis; ML, machine learning.

# Supplementary Figure 2 Radar Plot for the seven important predictors of severe PTS in 24 months


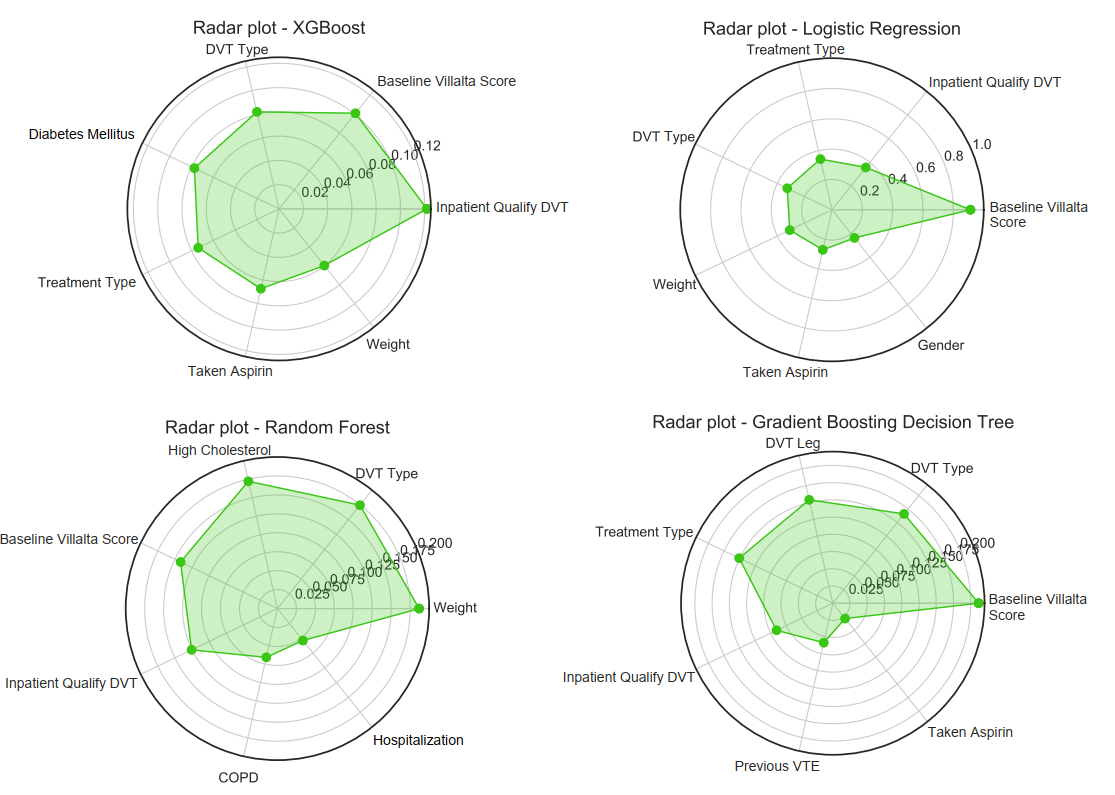


Higher value means more importance of the features determined by different ML algorithms.

Abbreviations: PTS, post-thrombotic syndrome; XGBoost, eXtreme gradient boosting; VTE, venous thromboembolism; BMI, body mass index; COPD, chronic obstructive pulmonary disease; DVT, deep vein thrombosis; ML, machine learning.

# Supplementary Table 5 Permutation importance for the 23 features of PTS in 24 months

| LR | | RF | | XGB | | GBDT | |
| --- | --- | --- | --- | --- | --- | --- | --- |
| Variables | Weight | Variables | Weight | Variables | Weight | Variables | Weight |
| Baseline Villalta Score | 0.464 | Weight | 0.194 | Diabetes Mellitus | 0.269 | Baseline Villalta Score | 0.503 |
| Diabetes Mellitus | 0.165 | Baseline Villalta Score | 0.137 | Baseline Villalta Score | 0.175 | Previous VTE | 0.148 |
| BMI | 0.154 | BMI | 0.134 | BMI | 0.163 | Diabetes Mellitus | 0.125 |
| Previous VTE | 0.132 | Diabetes Mellitus | 0.086 | Previous VTE | 0.138 | BMI | 0.100 |
| COPD | 0.093 | Inpatient Qualify DVT | 0.059 | High Cholesterol | 0.103 | Weight | 0.054 |
| Treatment Type | 0.009 | DVT Leg | 0.057 | Weight | 0.088 | High Cholesterol | 0.029 |
| High Cholesterol | 0.005 | Treatment Type | 0.050 | Treatment Type | 0.063 | Treatment Type | 0.026 |
| Weight | 0.000 | COPD | 0.042 | Major surgery | 0.000 | COPD | 0.013 |
| Height | 0.000 | Height | 0.041 | DVT Leg | 0.000 | Major surgery | 0.000 |
| Hypertension | 0.000 | Hypertension | 0.039 | Hospitalization | 0.000 | DVT Leg | 0.000 |
| DVT Leg | 0.000 | High Cholesterol | 0.038 | Plaster cast immob | 0.000 | Hospitalization | 0.000 |
| Age | 0.000 | Age | 0.028 | Taken Aspirin | 0.000 | Plaster cast immob | 0.000 |
| Hospitalization | 0.000 | Hospitalization | 0.022 | CHF | 0.000 | Taken Aspirin | 0.000 |
| Inpatient Qualify DVT | 0.000 | Previous VTE | 0.020 | angina or myocardial infarction | 0.000 | CHF | 0.000 |
| Major surgery | 0.000 | Major surgery | 0.019 | COPD | 0.000 | angina or myocardial infarction | 0.000 |
| CHF | 0.000 | CHF | 0.013 | Asthma | 0.000 | Asthma | 0.000 |
| angina or myocardial infarction | 0.000 | angina or myocardial infarction | 0.010 | Childbirth | 0.000 | Childbirth | 0.000 |
| Childbirth | 0.000 | Childbirth | 0.007 | Inpatient Qualify DVT | 0.000 | Inpatient Qualify DVT | 0.000 |
| DVT Type | 0.000 | DVT Type | 0.003 | Hypertension | 0.000 | Hypertension | 0.000 |
| Taken Aspirin | 0.000 | Taken Aspirin | 0.002 | Gender | 0.000 | Gender | 0.000 |
| Gender | 0.000 | Gender | 0.000 | Age | 0.000 | Age | 0.000 |
| Plaster cast immob | 0.000 | Plaster cast immob | 0.000 | DVT Type | 0.000 | DVT Type | 0.000 |
| Asthma | 0.000 | Asthma | 0.000 | Height | 0.000 | Height | 0.000 |

A feature is considered to be “important” if its weight value is high; a feature is considered to be “unimportant” if its weight value is low or zero.

Abbreviations: BMI, body mass index; DVT, deep vein thrombosis; VTE, venous thromboembolism; COPD, chronic obstructive pulmonary disease; CHF, congestive heart failure; PTS, post-thrombotic syndrome; RF, random forest; LR, logistic regression; GBDT, gradient boosting decision tree; XGB, extreme gradient boosting.

# Supplementary Table 6 Permutation importance for the 23 features of moderate-severe PTS in 24 months

| LR | | RF | | XGB | | GBDT | |
| --- | --- | --- | --- | --- | --- | --- | --- |
| Variables | Weight | Variables | Weight | Variables | Weight | Variables | Weight |
| Baseline Villalta Score | 0.758 | DVT Type | 0.251 | Diabetes Mellitus | 0.18 | Baseline Villalta Score | 0.408 |
| Diabetes Mellitus | 0.251 | Baseline Villalta Score | 0.142 | Baseline Villalta Score | 0.142 | Diabetes Mellitus | 0.221 |
| Treatment Type | 0.246 | Diabetes Mellitus | 0.104 | Treatment Type | 0.089 | Treatment Type | 0.177 |
| Gender | 0.113 | Treatment Type | 0.089 | DVT Leg | 0.062 | DVT Type | 0.076 |
| BMI | 0.108 | Hypertension | 0.085 | Previous VTE | 0.057 | Age | 0.031 |
| Inpatient Qualify DVT | 0.091 | Inpatient Qualify DVT | 0.066 | BMI | 0.057 | Hypertension | 0.03 |
| angina or myocardial infarction | 0.066 | BMI | 0.053 | DVT Type | 0.056 | DVT Leg | 0.016 |
| Age | 0.063 | DVT Leg | 0.036 | Inpatient Qualify DVT | 0.054 | Inpatient Qualify DVT | 0.015 |
| COPD | 0.061 | High Cholesterol | 0.03 | High Cholesterol | 0.05 | Taken Aspirin | 0.013 |
| DVT Type | 0.058 | Taken Aspirin | 0.030 | Hypertension | 0.048 | Previous VTE | 0.013 |
| Childbirth | 0.013 | angina or myocardial infarction | 0.027 | Taken Aspirin | 0.045 | Childbirth | 0.000 |
| DVT Leg | -0.011 | Weight | 0.018 | Age | 0.043 | Asthma | 0.000 |
| Taken Aspirin | -0.021 | Asthma | 0.015 | COPD | 0.041 | Gender | 0.000 |
| Plaster cast immob | -0.047 | Previous VTE | 0.014 | Gender | 0.040 | High Cholesterol | 0.000 |
| Height | 0.000 | COPD | 0.013 | Weight | 0.020 | angina or myocardial infarction | 0.000 |
| Hypertension | 0.000 | Height | 0.010 | angina or myocardial infarction | 0.016 | COPD | 0.000 |
| High Cholesterol | 0.000 | Age | 0.007 | Childbirth | 0.000 | Plaster cast immob | 0.000 |
| Major surgery | 0.000 | Gender | 0.007 | Asthma | 0.000 | CHF | 0.000 |
| Hospitalization | 0.000 | Major surgery | 0.002 | Plaster cast immob | 0.000 | Weight | 0.000 |
| Weight | 0.000 | Hospitalization | 0.000 | CHF | 0.000 | BMI | 0.000 |
| CHF | 0.000 | Childbirth | 0.000 | Major surgery | 0.000 | Major surgery | 0.000 |
| Asthma | 0.000 | CHF | 0.000 | Hospitalization | 0.000 | Hospitalization | 0.000 |
| Previous VTE | 0.000 | Plaster cast immob | 0.000 | Height | 0.000 | Height | 0.000 |

A feature is considered to be “important” if its weight value is high; a feature is considered to be “unimportant” if its weight value is low or zero.

Abbreviations: BMI, body mass index; DVT, deep vein thrombosis; VTE, venous thromboembolism; COPD, chronic obstructive pulmonary disease; CHF, congestive heart failure; PTS, post-thrombotic syndrome; RF, random forest; LR, logistic regression; GBDT, gradient boosting decision tree; XGB, extreme gradient boosting.

# Supplementary Table 7 Permutation importance for the 23 features of severe PTS in 24 months.

| LR | | RF | | XGB | | GBDT | |
| --- | --- | --- | --- | --- | --- | --- | --- |
| Variables | Weight | Variables | Weight | Variables | Weight | Variables | Weight |
| Baseline Villalta Score | 0.912 | Weight | 0.187 | Inpatient Qualify DVT | 0.122 | Baseline Villalta Score | 0.211 |
| Inpatient Qualify DVT | 0.357 | DVT Type | 0.174 | Baseline Villalta Score | 0.101 | DVT Type | 0.166 |
| Treatment Type | 0.343 | High Cholesterol | 0.172 | DVT Type | 0.082 | DVT Leg | 0.154 |
| DVT Type | 0.327 | Baseline Villalta Score | 0.142 | Diabetes Mellitus | 0.077 | Treatment Type | 0.151 |
| Weight | 0.309 | Inpatient Qualify DVT | 0.126 | Treatment Type | 0.074 | Inpatient Qualify DVT | 0.091 |
| Gender | 0.237 | COPD | 0.066 | Taken Aspirin | 0.068 | Previous VTE | 0.059 |
| angina or myocardial infarction | 0.140 | Hospitalization | 0.054 | Weight | 0.060 | Taken Aspirin | 0.028 |
| Plaster cast immob | 0.103 | CHF | 0.035 | Gender | 0.058 | Weight | 0.028 |
| COPD | 0.073 | Diabetes Mellitus | 0.018 | DVT Leg | 0.055 | Hospitalization | 0.025 |
| BMI | 0.068 | Age | 0.015 | Previous VTE | 0.048 | Age | 0.022 |
| Age | 0.043 | Plaster cast immob | 0.008 | Hospitalization | 0.045 | Height | 0.017 |
| Major surgery | 0.029 | angina or myocardial infarction | 0.003 | Height | 0.035 | Diabetes Mellitus | 0.017 |
| Diabetes Mellitus | 0.016 | Asthma | 0.000 | Major surgery | 0.030 | Hypertension | 0.016 |
| Hypertension | -0.048 | Gender | 0.000 | Hypertension | 0.028 | Gender | 0.016 |
| CHF | -0.100 | Hypertension | 0.000 | angina or myocardial infarction | 0.027 | High Cholesterol | 0.000 |
| Asthma | -0.137 | Height | 0.000 | Age | 0.027 | Plaster cast immob | 0.000 |
| Hospitalization | -0.163 | Taken Aspirin | 0.000 | BMI | 0.025 | Asthma | 0.000 |
| DVT Leg | -0.191 | BMI | 0.000 | High Cholesterol | 0.022 | COPD | 0.000 |
| Childbirth | -0.194 | DVT Leg | 0.000 | Plaster cast immob | 0.016 | CHF | 0.000 |
| Taken Aspirin | -0.271 | Previous VTE | 0.000 | CHF | 0.000 | BMI | 0.000 |
| Height | 0.000 | Major surgery | 0.000 | Asthma | 0.000 | Childbirth | 0.000 |
| High Cholesterol | 0.000 | Childbirth | 0.000 | Childbirth | 0.000 | Major surgery | 0.000 |
| Previous VTE | 0.000 | Treatment Type | 0.000 | COPD | 0.000 | angina or myocardial infarction | 0.000 |

A feature is considered to be “important” if its weight value is high; a feature is considered to be “unimportant” if its weight value is low or zero.

Abbreviations: BMI, body mass index; DVT, deep vein thrombosis; VTE, venous thromboembolism; COPD, chronic obstructive pulmonary disease; CHF, congestive heart failure; PTS, post-thrombotic syndrome; RF, random forest; LR, logistic regression; GBDT, gradient boosting decision tree; XGB, extreme gradient boosting.

# Supplementary Figure 3 PDP for the 23 features of PTS in 24 months

**(a) PDP of PTS on 23 features, with XGB**


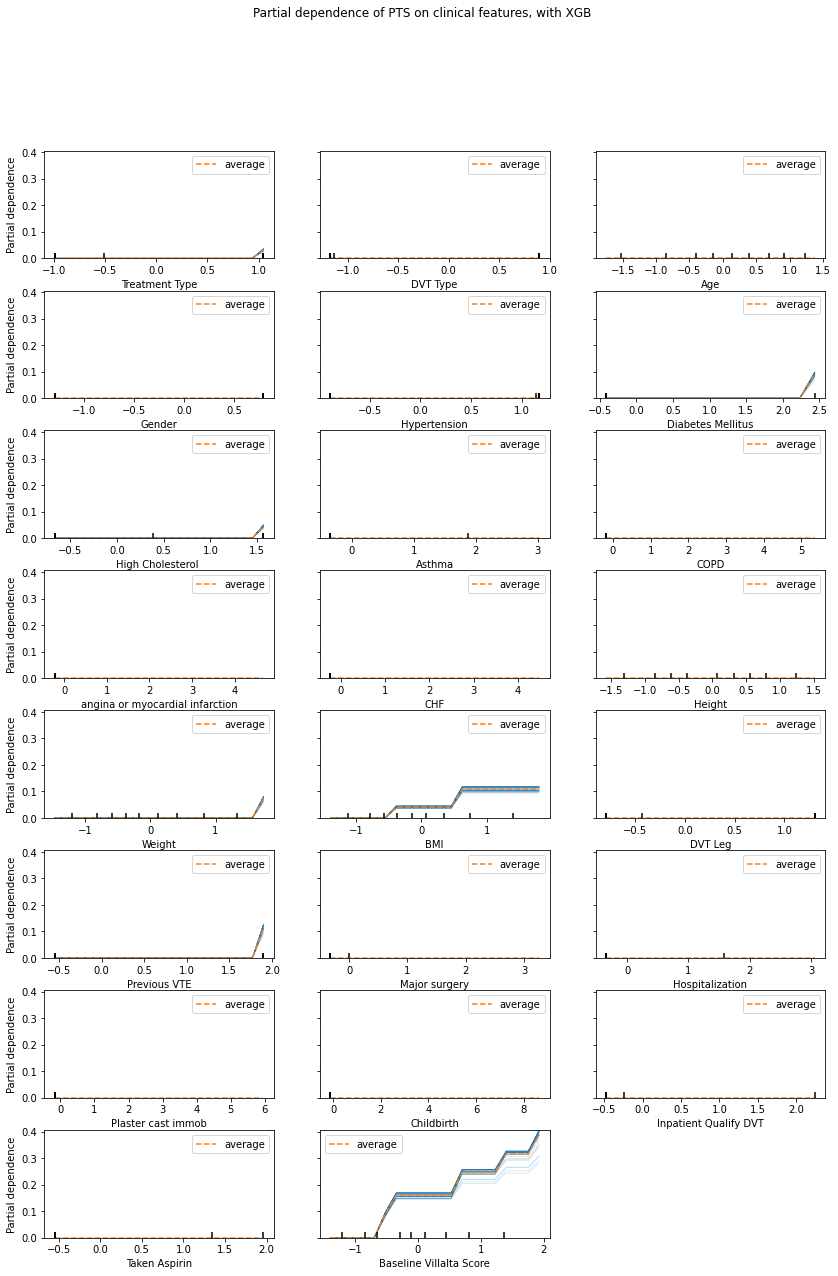


The influence of each feature on the predicted target is visualized in the PDP.

Abbreviations: Partial Dependence Plots; BMI, body mass index; DVT, deep vein thrombosis; VTE, venous thromboembolism; COPD, chronic obstructive pulmonary disease; CHF, congestive heart failure; PTS, post-thrombotic syndrome; XGB, extreme gradient boosting.

**(b) PDP of PTS on 23 features, with LR**


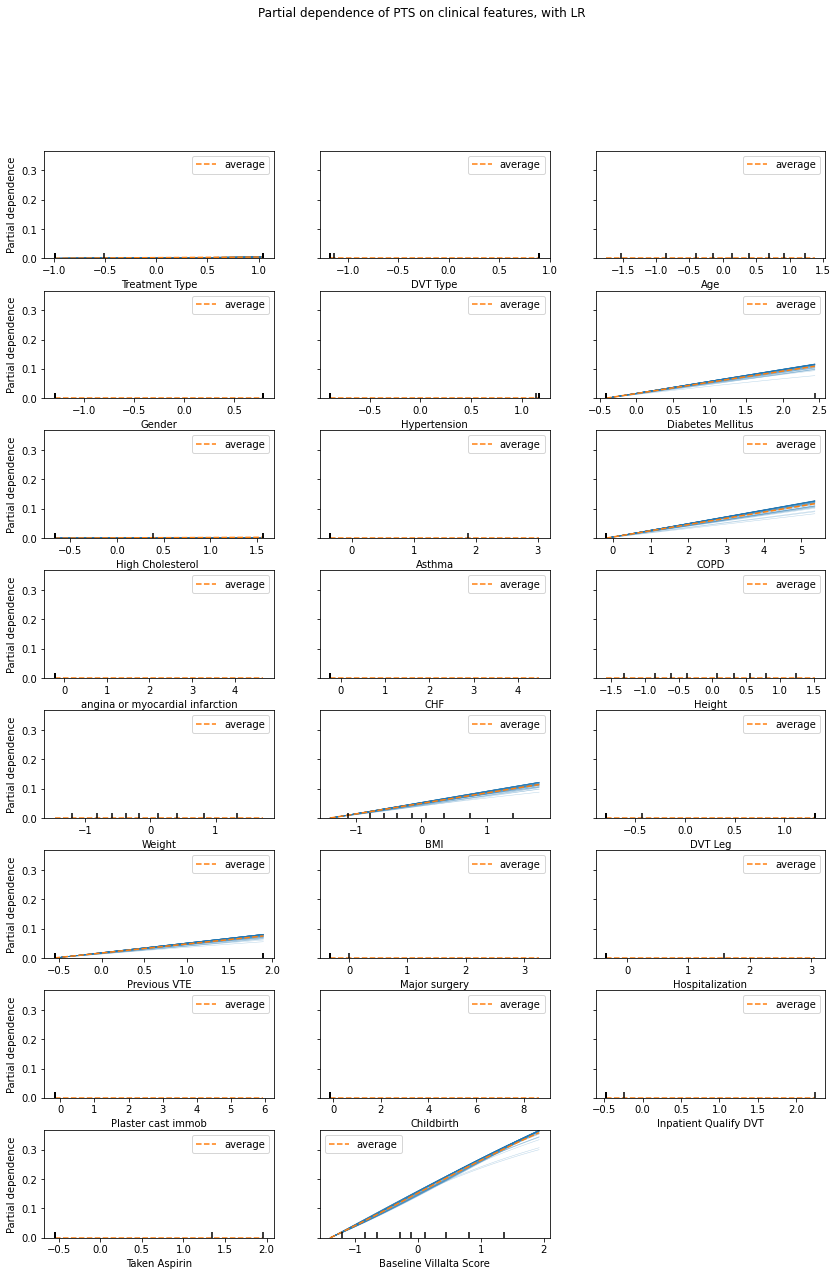


The influence of each feature on the predicted target is visualized in the PDP.

Abbreviations: Partial Dependence Plots; BMI, body mass index; DVT, deep vein thrombosis; VTE, venous thromboembolism; COPD, chronic obstructive pulmonary disease; CHF, congestive heart failure; PTS, post-thrombotic syndrome; LR, logistic regression.

**(c) PDP of PTS on 23 features, with RF**


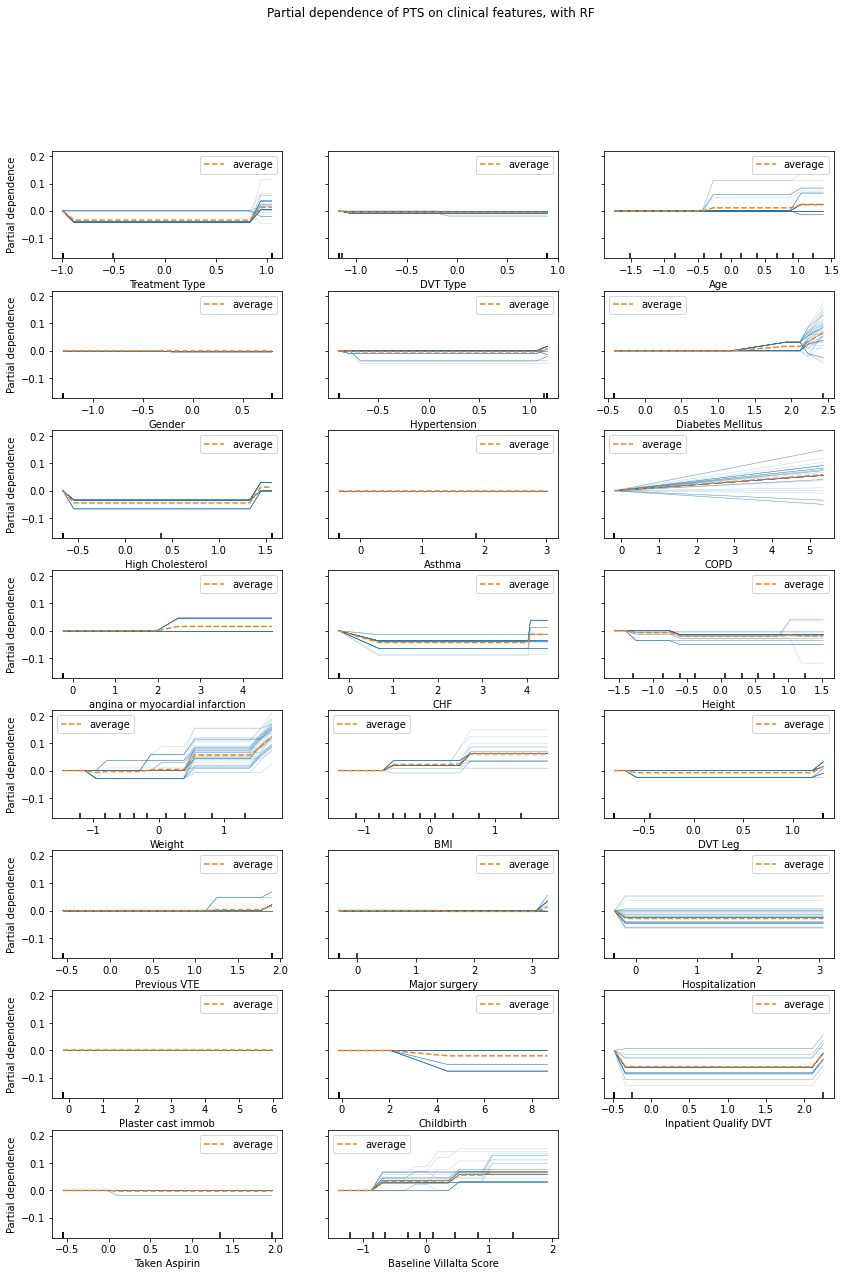


The influence of each feature on the predicted target is visualized in the PDP.

Abbreviations: Partial Dependence Plots; BMI, body mass index; DVT, deep vein thrombosis; VTE, venous thromboembolism; COPD, chronic obstructive pulmonary disease; CHF, congestive heart failure; PTS, post-thrombotic syndrome; RF, random forest.

**(d) PDP of PTS on 23 features, with GBDT**


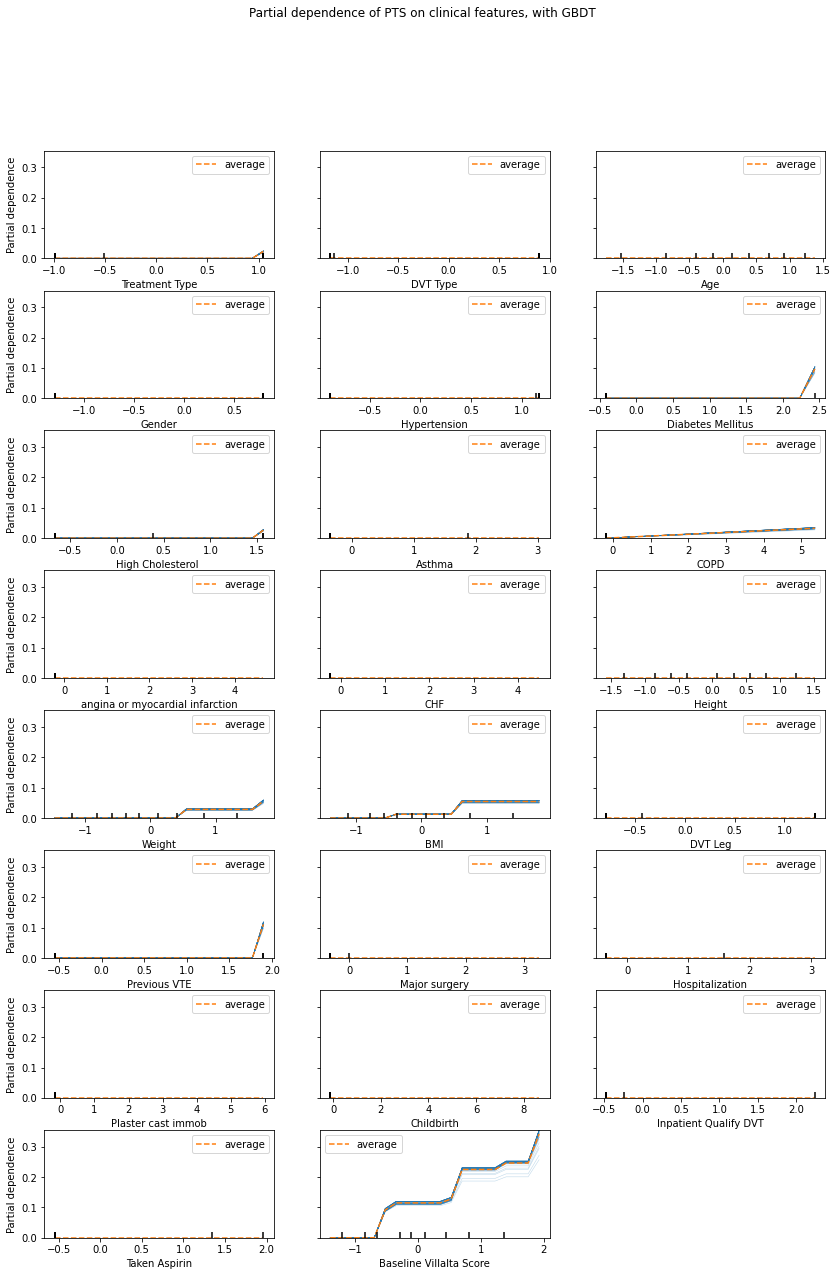


The influence of each feature on the predicted target is visualized in the PDP.

Abbreviations: Partial Dependence Plots; BMI, body mass index; DVT, deep vein thrombosis; VTE, venous thromboembolism; COPD, chronic obstructive pulmonary disease; CHF, congestive heart failure; PTS, post-thrombotic syndrome; GBDT, gradient boosting decision tree.

# Supplementary Figure 4 PDP for the 23 features of moderate-severe PTS in 24 months

**(a) PDP of moderate-severe PTS on 23 features, with XGB**


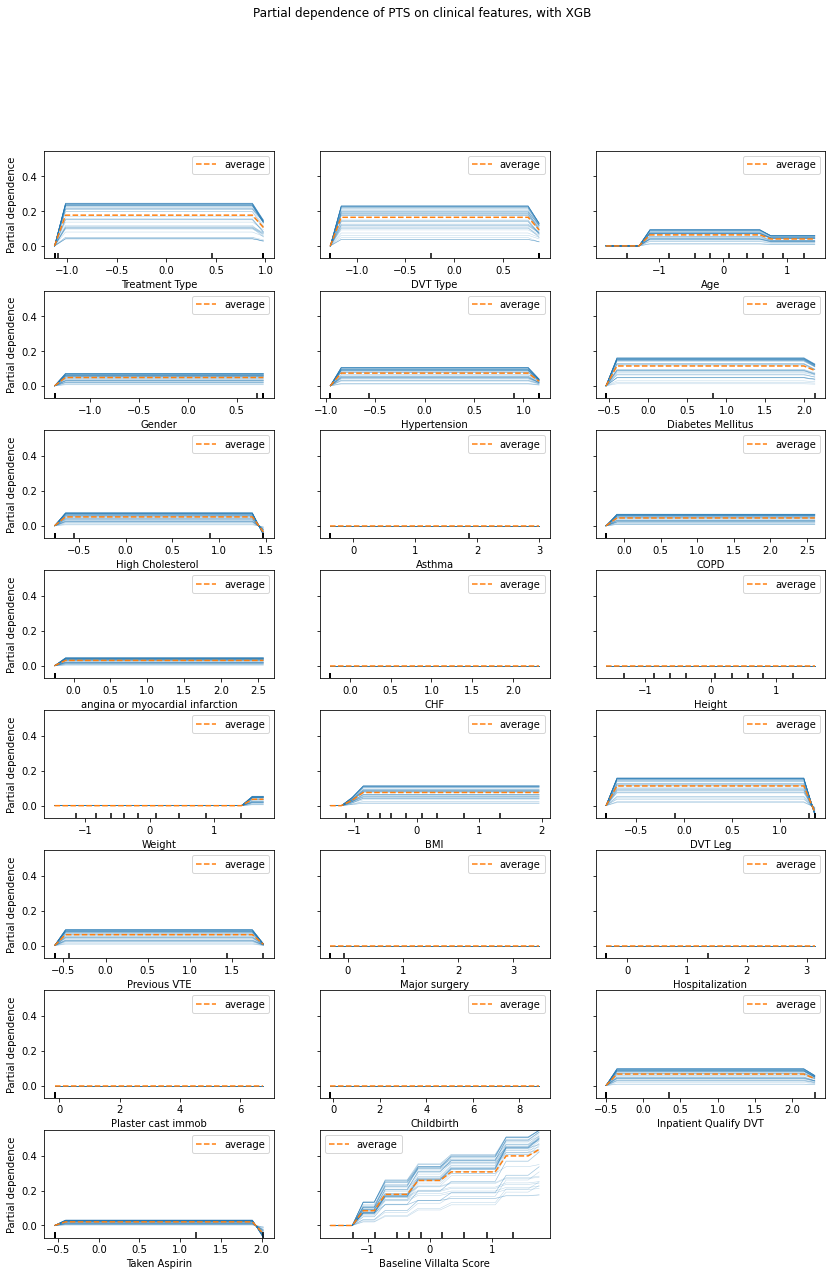


The influence of each feature on the predicted target is visualized in the PDP.

Abbreviations: Partial Dependence Plots; BMI, body mass index; DVT, deep vein thrombosis; VTE, venous thromboembolism; COPD, chronic obstructive pulmonary disease; CHF, congestive heart failure; PTS, post-thrombotic syndrome; XGB, extreme gradient boosting.

**(b) PDP of moderate-severe PTS on 23 features, with LR**


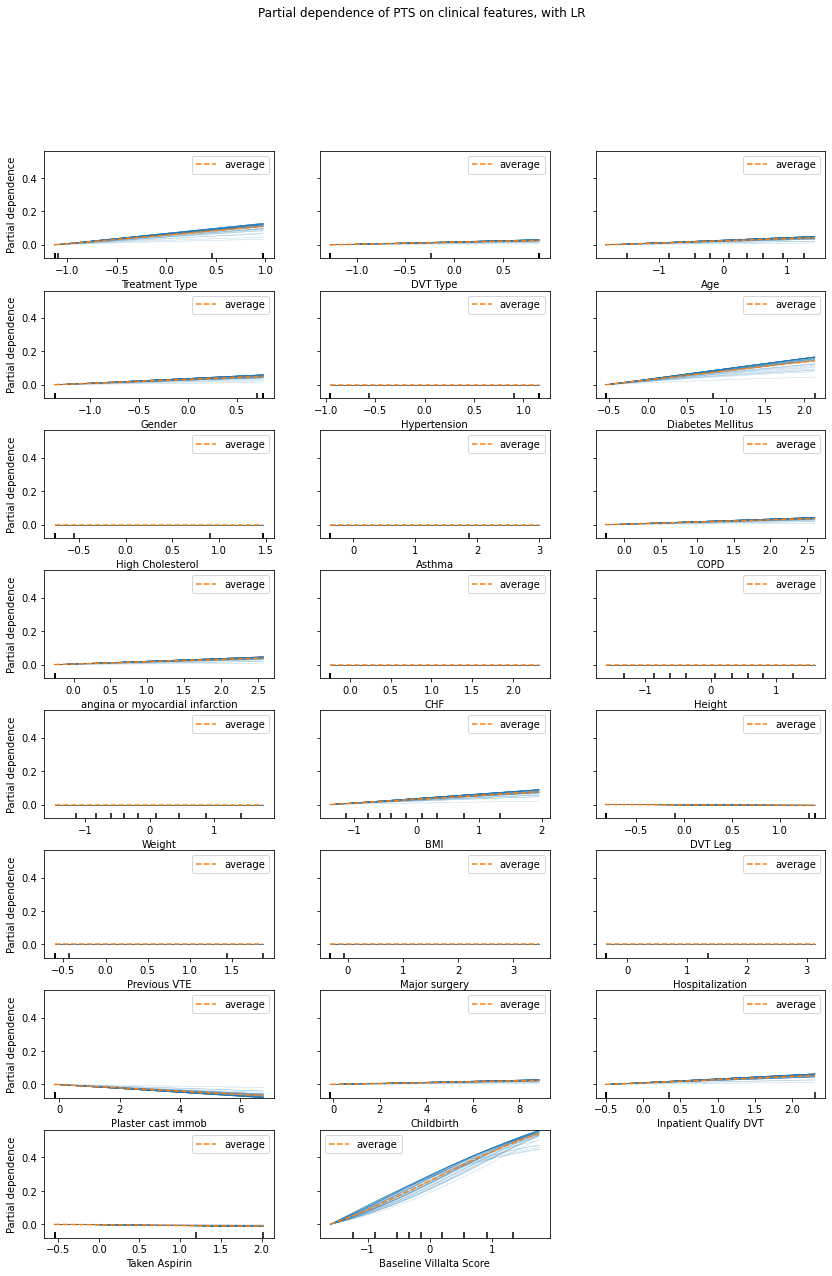


The influence of each feature on the predicted target is visualized in the PDP.

Abbreviations: Partial Dependence Plots; BMI, body mass index; DVT, deep vein thrombosis; VTE, venous thromboembolism; COPD, chronic obstructive pulmonary disease; CHF, congestive heart failure; PTS, post-thrombotic syndrome; LR, logistic regression.

**(c) PDP of moderate-severe PTS on 23 features, with RF**


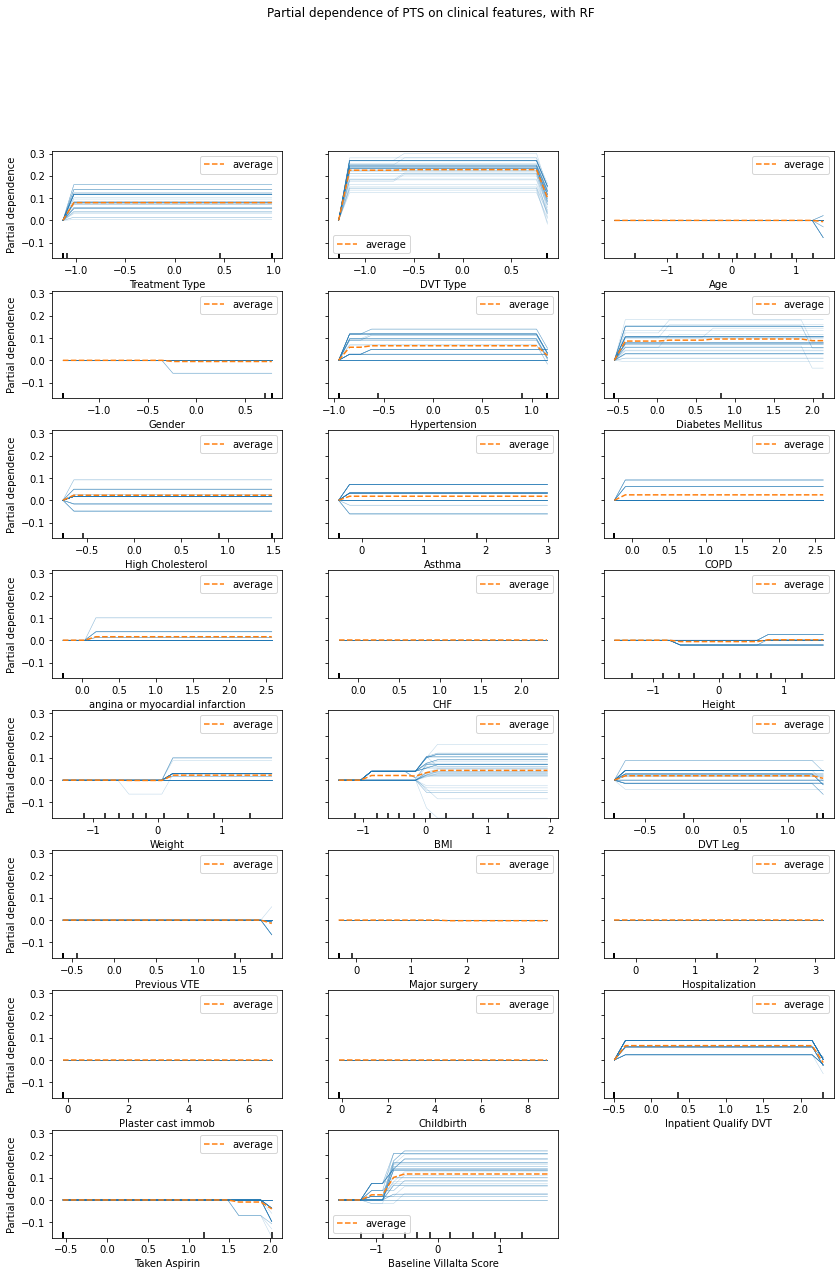


The influence of each feature on the predicted target is visualized in the PDP.

Abbreviations: Partial Dependence Plots; BMI, body mass index; DVT, deep vein thrombosis; VTE, venous thromboembolism; COPD, chronic obstructive pulmonary disease; CHF, congestive heart failure; PTS, post-thrombotic syndrome; RF, random forest.

**(d) PDP of moderate-severe PTS on 23 features, with GBDT**


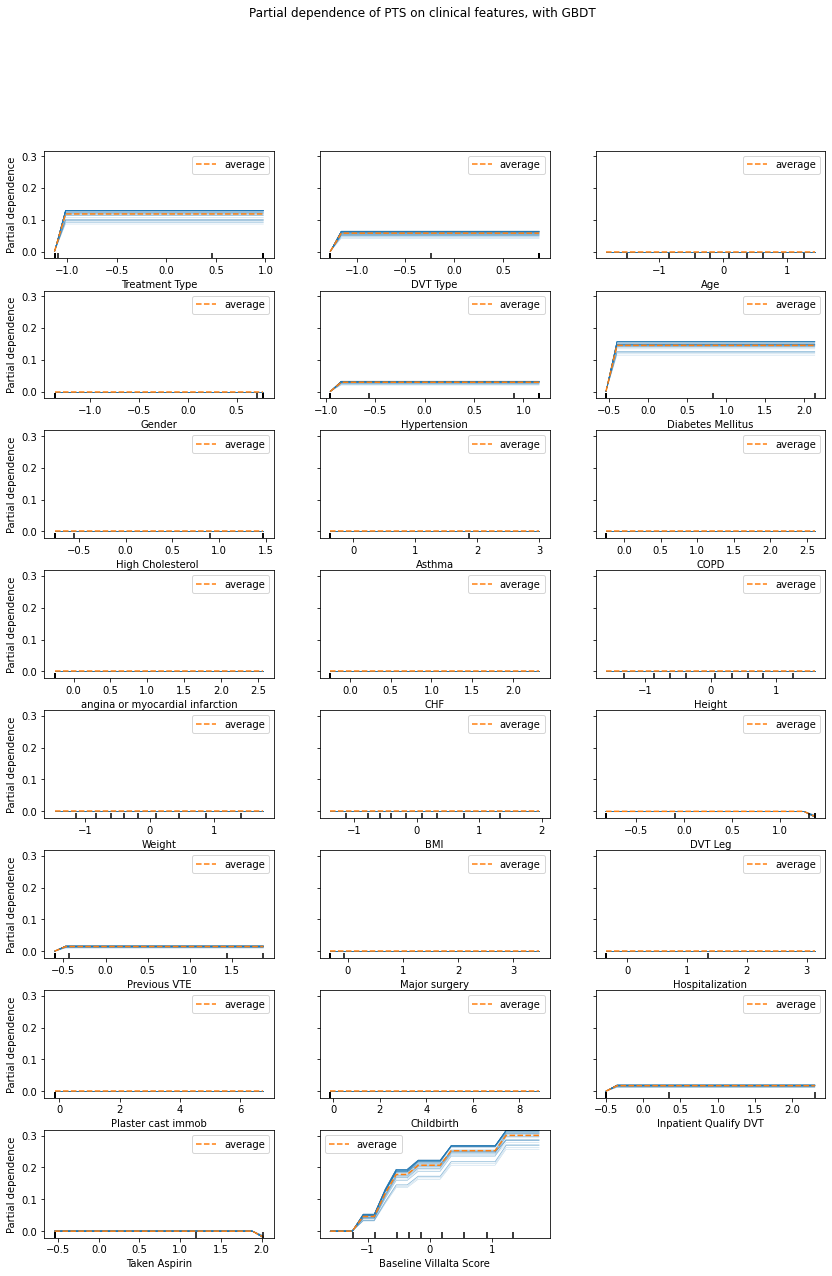


The influence of each feature on the predicted target is visualized in the PDP.

Abbreviations: Partial Dependence Plots; BMI, body mass index; DVT, deep vein thrombosis; VTE, venous thromboembolism; COPD, chronic obstructive pulmonary disease; CHF, congestive heart failure; PTS, post-thrombotic syndrome; GBDT, gradient boosting decision tree.

# Supplementary Figure 5 PDP for the 23 features of severe PTS in 24 months

**(a) PDP of severe PTS on 23 features, with XGB**


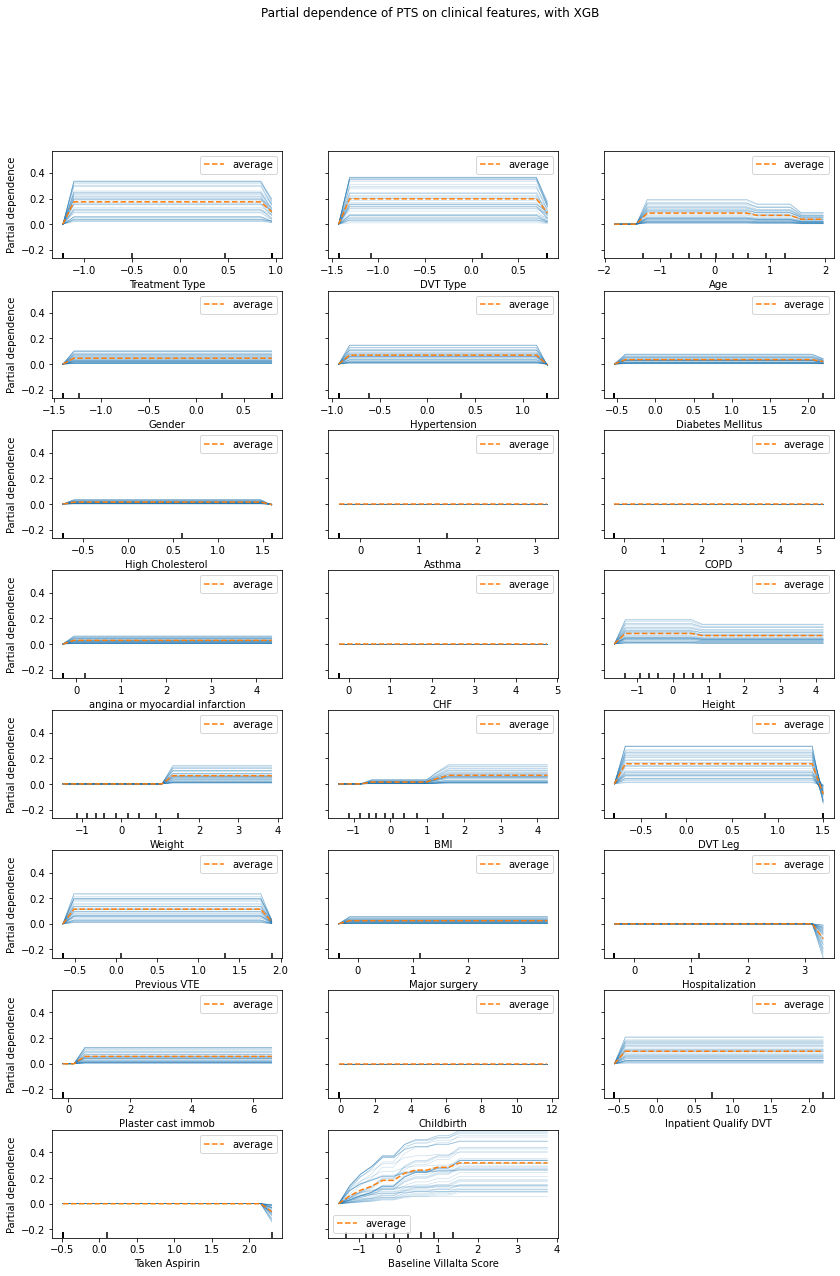


The influence of each feature on the predicted target is visualized in the PDP.

Abbreviations: Partial Dependence Plots; BMI, body mass index; DVT, deep vein thrombosis; VTE, venous thromboembolism; COPD, chronic obstructive pulmonary disease; CHF, congestive heart failure; PTS, post-thrombotic syndrome; XGB, extreme gradient boosting.

**(b) PDP of severe PTS on 23 features, with LR**


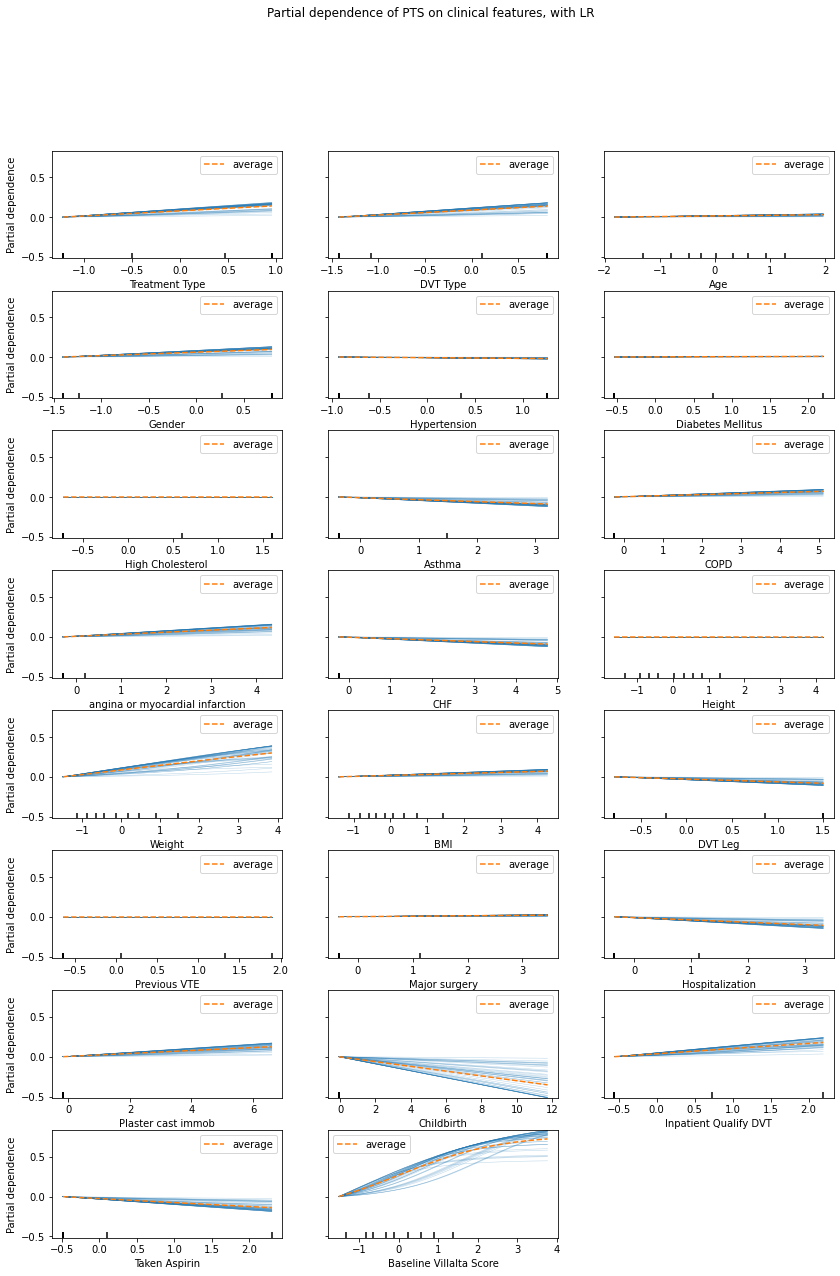


The influence of each feature on the predicted target is visualized in the PDP.

Abbreviations: Partial Dependence Plots; BMI, body mass index; DVT, deep vein thrombosis; VTE, venous thromboembolism; COPD, chronic obstructive pulmonary disease; CHF, congestive heart failure; PTS, post-thrombotic syndrome; LR, logistic regression.

**(c) PDP of severe PTS on 23 features, with RF**


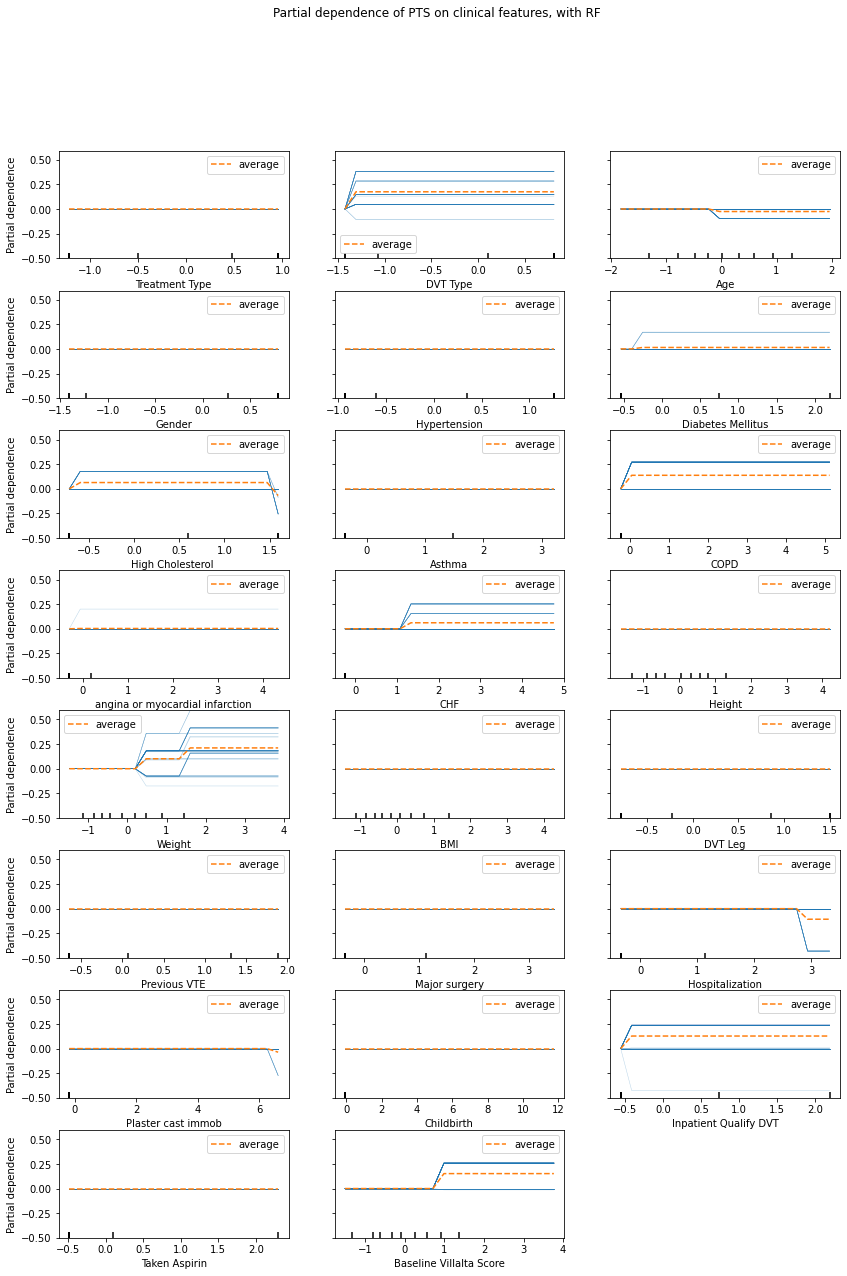


The influence of each feature on the predicted target is visualized in the PDP.

Abbreviations: Partial Dependence Plots; BMI, body mass index; DVT, deep vein thrombosis; VTE, venous thromboembolism; COPD, chronic obstructive pulmonary disease; CHF, congestive heart failure; PTS, post-thrombotic syndrome; RF, random forest.

**(d) PDP of severe PTS on 23 features, with GBDT**


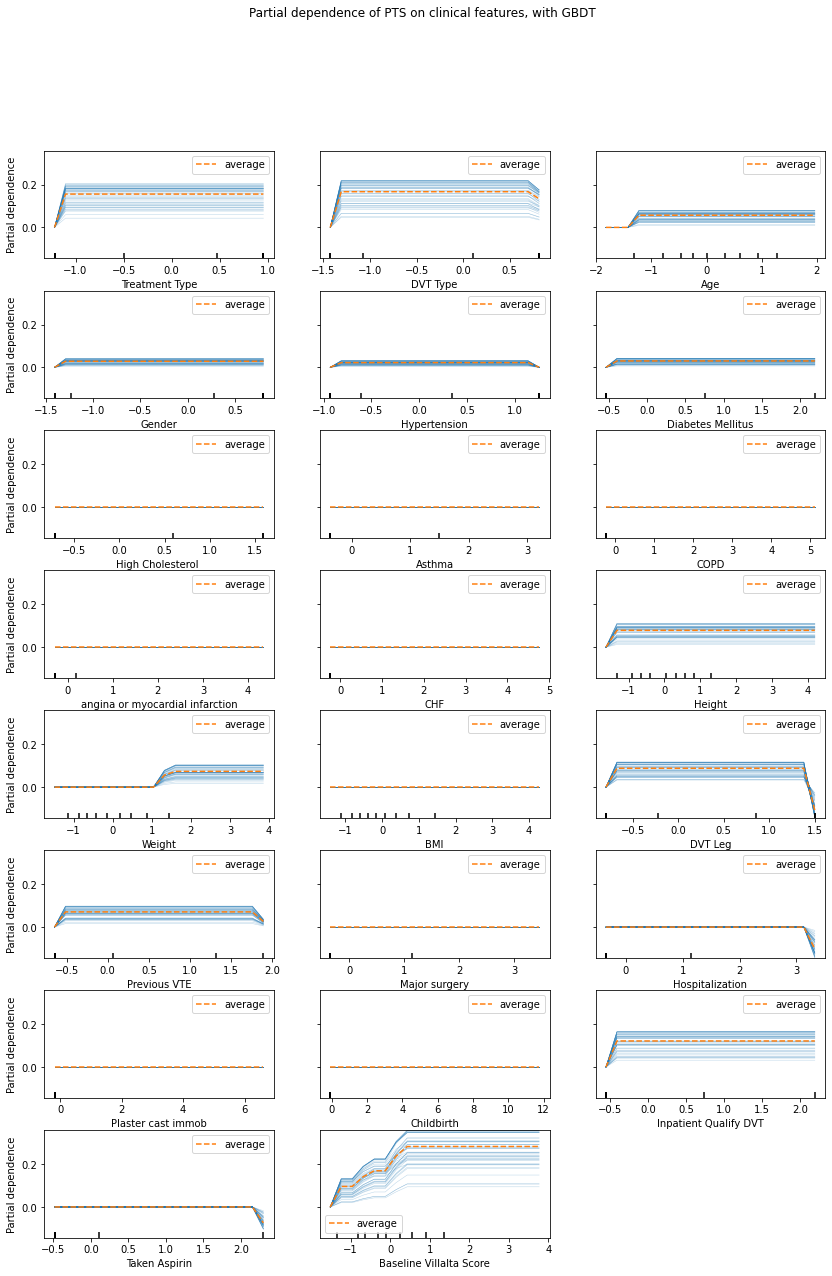


The influence of each feature on the predicted target is visualized in the PDP.

Abbreviations: Partial Dependence Plots; BMI, body mass index; DVT, deep vein thrombosis; VTE, venous thromboembolism; COPD, chronic obstructive pulmonary disease; CHF, congestive heart failure; PTS, post-thrombotic syndrome; GBDT, gradient boosting decision tree.

# Supplementary Figure 6 The SHAP force plot of PTS in 24 months

1. **The SHAP force plot of PTS in 24 months with XGB**

**
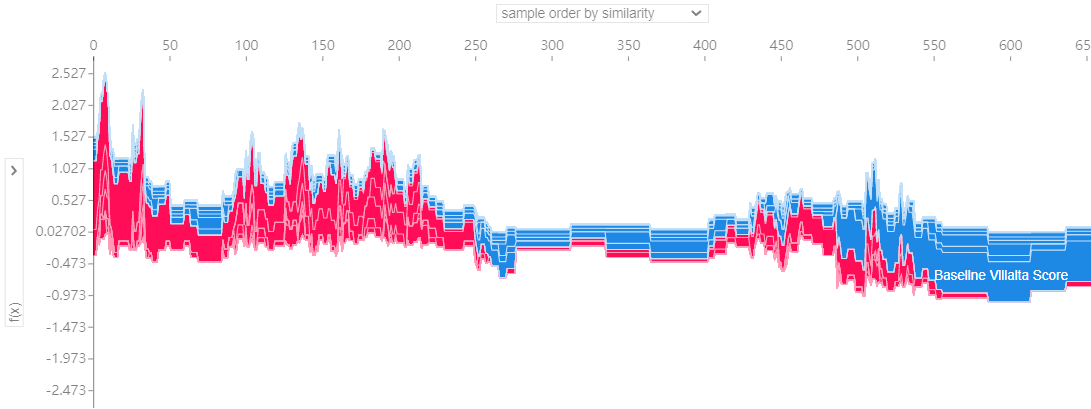
**

1. **The SHAP force plot of PTS in 24 months with LR**

**
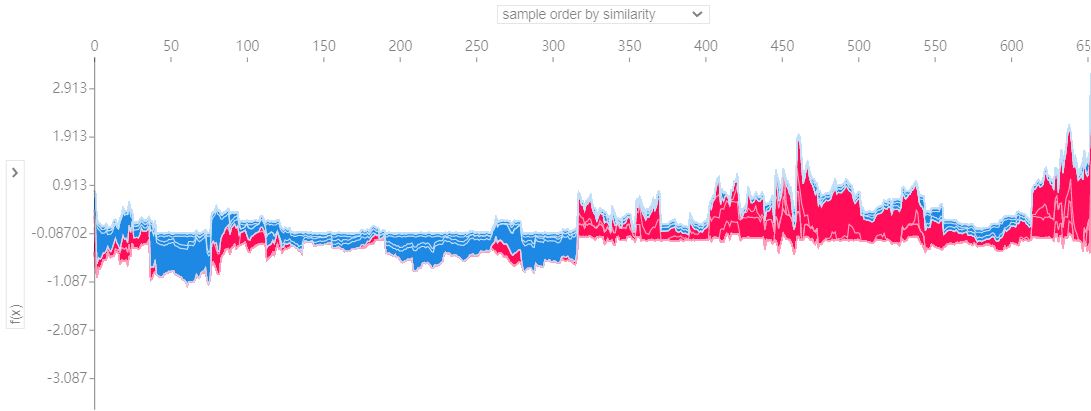
**

1. **The SHAP force plot of PTS in 24 months with RF**

**
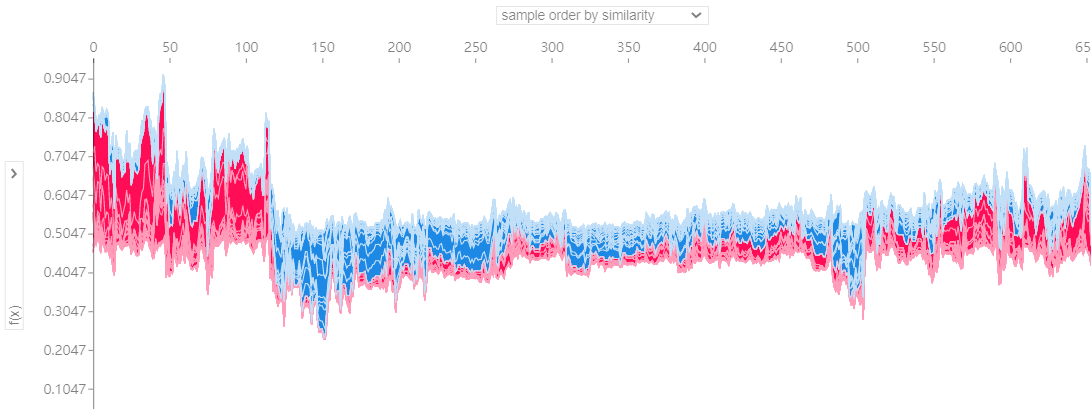
**

1. **The SHAP force plot of PTS in 24 months with GBDT**


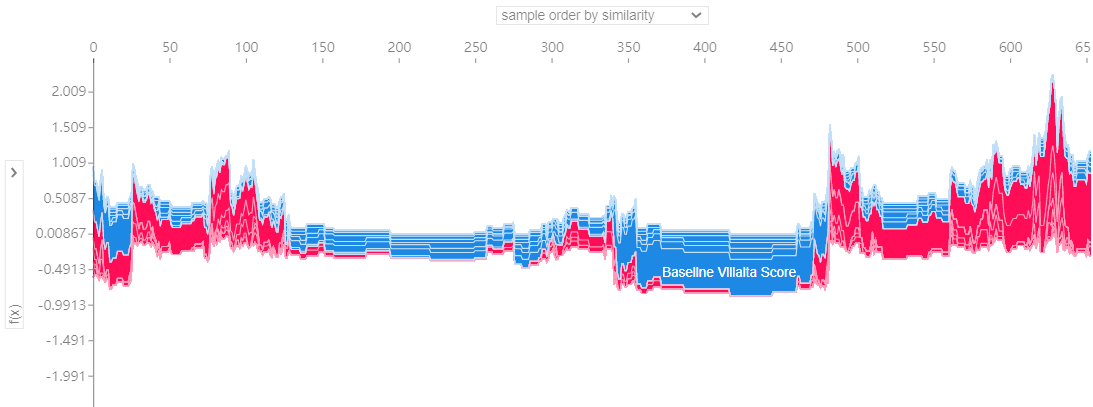


The SHAP force plot shows which features has the most influence on the model’s prediction for a single observation. Each vertical line in the longitudinal direction represents the feature importances of a single observation. And all observations stack horizontally to constitute force plot. Each observation has its special model’s score in the plot. Higher scores lead the model to predict 1 and lower scores lead to predict 0. The features, which are important to making the prediction for this observation, are shown in red and blue, with blue representing features that pushed the score lower, and red representing features that pushed the score higher. The features that have a greater impact on the score are located closer to the boundary between red and blue.

Abbreviations: SHAP, SHapley Additive exPlanations; PTS, post-thrombotic syndrome; RF, random forest; LR, logistic regression; GBDT, gradient boosting decision tree; XGB, extreme gradient boosting.

# Supplementary Figure 7 The SHAP force plot of moderate-severe PTS in 24 months

1. **The SHAP force plot of moderate-severe PTS in 24 months with XGB**

**
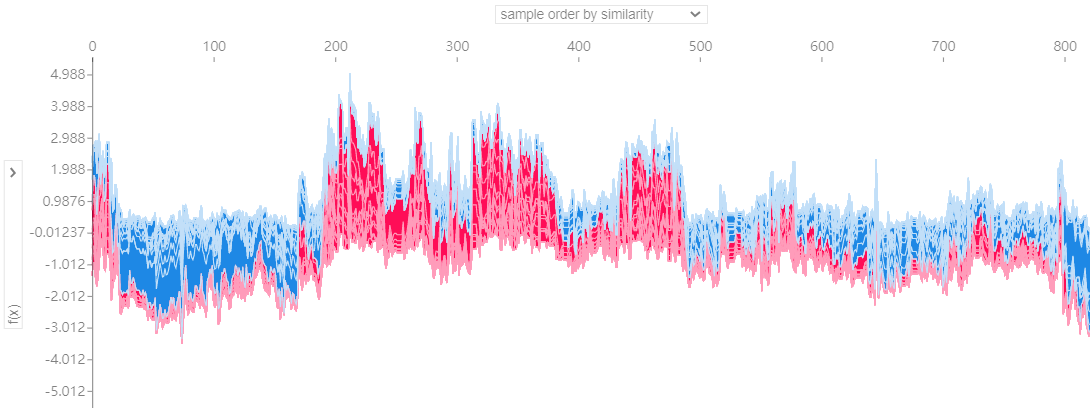
**

1. **The SHAP force plot of moderate-severe PTS in 24 months with LR**

**
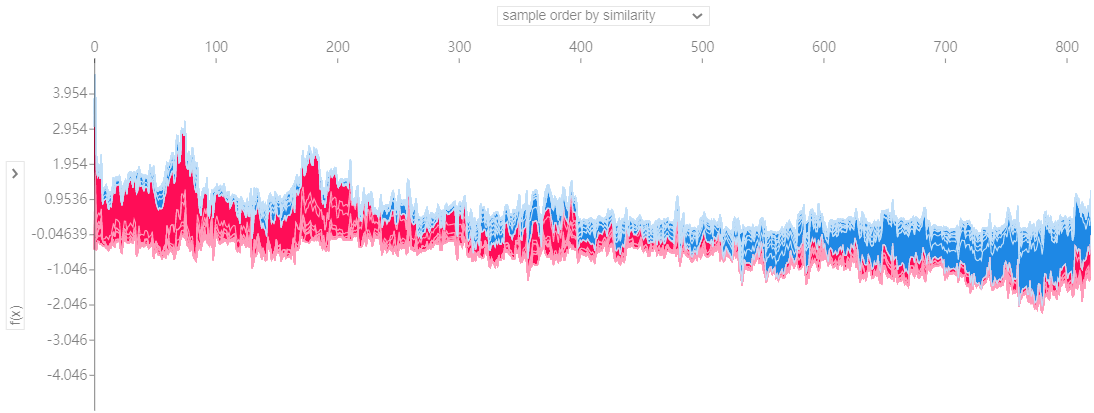
**

1. **The SHAP force plot of moderate-severe PTS in 24 months with RF**

**
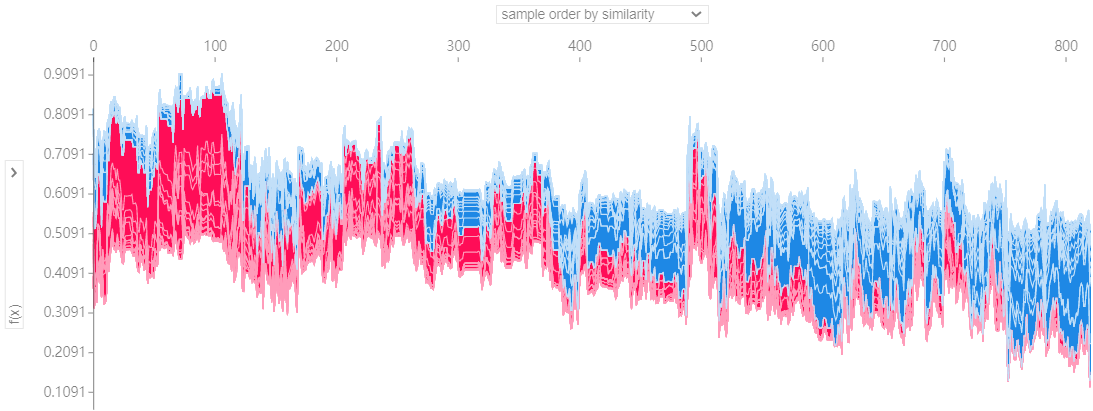
**

1. **The SHAP force plot of moderate-severe PTS in 24 months with GBDT**


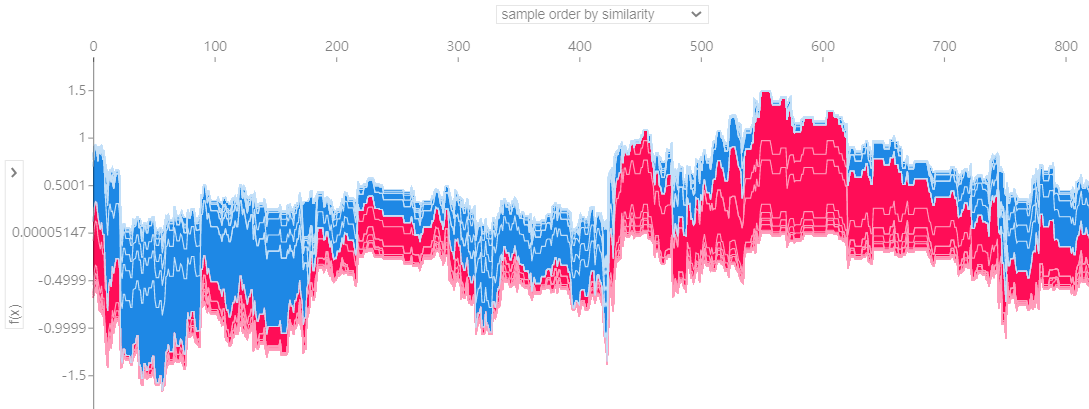


The SHAP force plot shows which features has the most influence on the model’s prediction for a single observation. Each vertical line in the longitudinal direction represents the feature importances of a single observation. And all observations stack horizontally to constitute force plot. Each observation has its special model’s score in the plot. Higher scores lead the model to predict 1 and lower scores lead to predict 0. The features, which are important to making the prediction for this observation, are shown in red and blue, with blue representing features that pushed the score lower, and red representing features that pushed the score higher. The features that have a greater impact on the score are located closer to the boundary between red and blue.

Abbreviations: SHAP, SHapley Additive exPlanations; PTS, post-thrombotic syndrome; RF, random forest; LR, logistic regression; GBDT, gradient boosting decision tree; XGB, extreme gradient boosting.

# Supplementary Figure 8 The SHAP force plot of severe PTS in 24 months

1. **The SHAP force plot of severe PTS in 24 months with XGB**

**
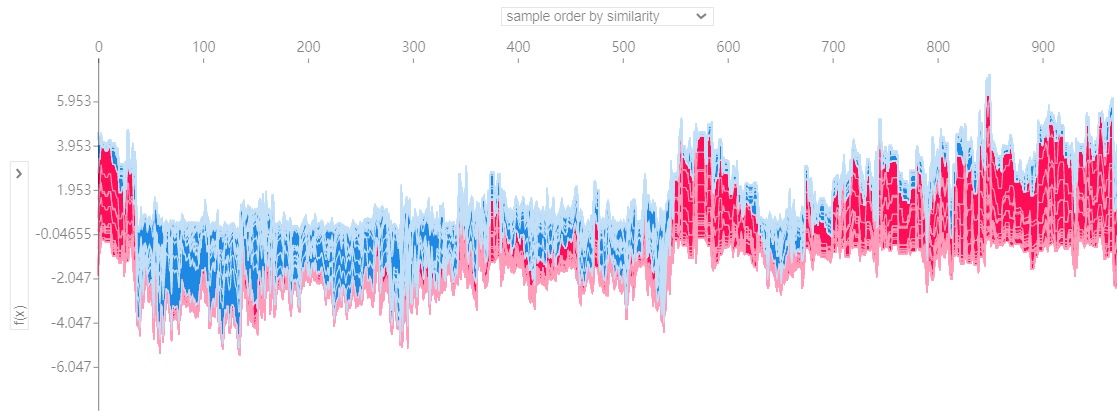
**

1. **The SHAP force plot of severe PTS in 24 months with LR**

**
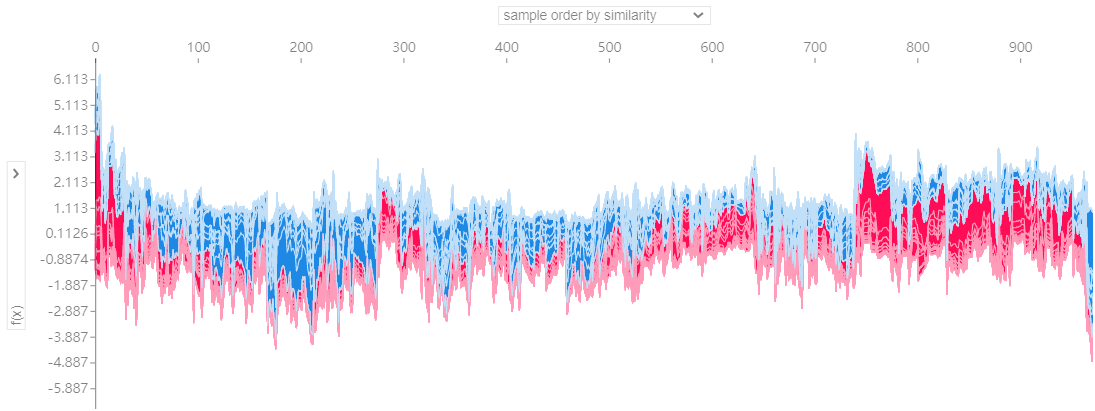
**

1. **The SHAP force plot of severe PTS in 24 months with RF**

**
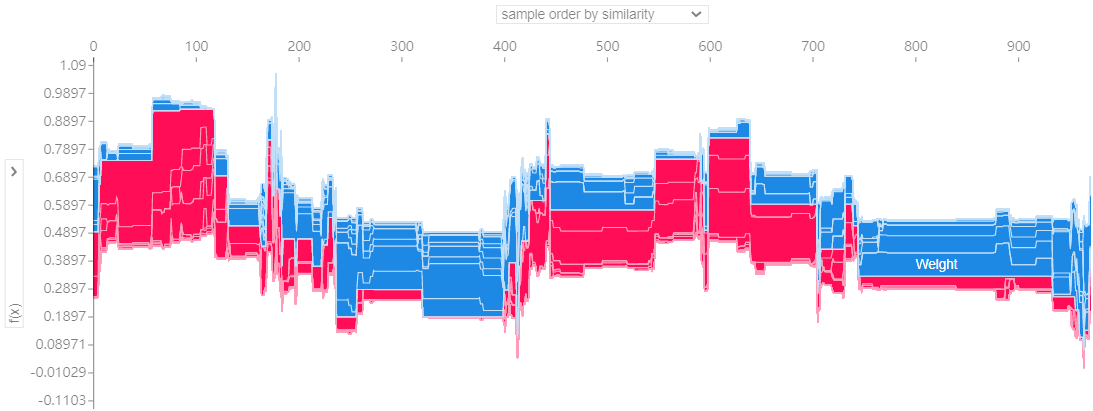
**

1. **The SHAP force plot of severe PTS in 24 months with GBDT**


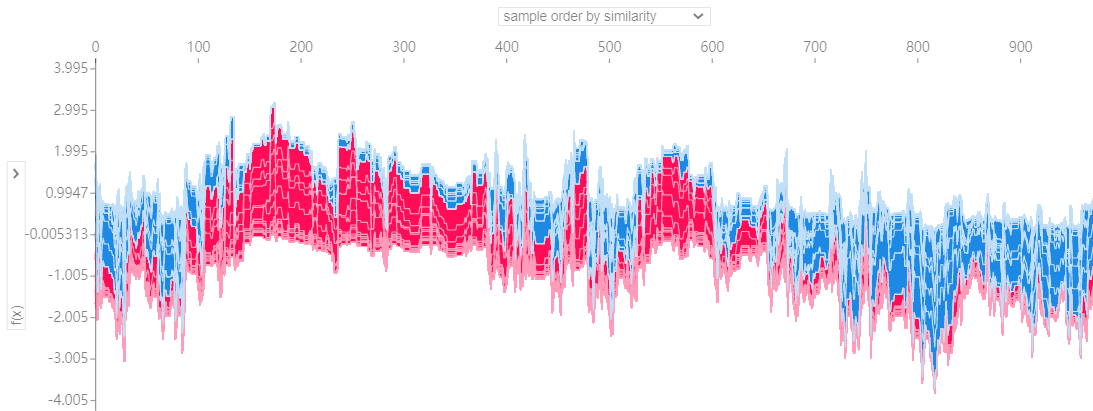


The SHAP force plot shows which features has the most influence on the model’s prediction for a single observation. Each vertical line in the longitudinal direction represents the feature importances of a single observation. And all observations stack horizontally to constitute force plot. Each observation has its special model’s score in the plot. Higher scores lead the model to predict 1 and lower scores lead to predict 0. The features, which are important to making the prediction for this observation, are shown in red and blue, with blue representing features that pushed the score lower, and red representing features that pushed the score higher. The features that have a greater impact on the score are located closer to the boundary between red and blue.

Abbreviations: SHAP, SHapley Additive exPlanations; PTS, post-thrombotic syndrome; RF, random forest; LR, logistic regression; GBDT, gradient boosting decision tree; XGB, extreme gradient boosting.

# Supplementary Figure 9 The feature importance plot of PTS in 24 months


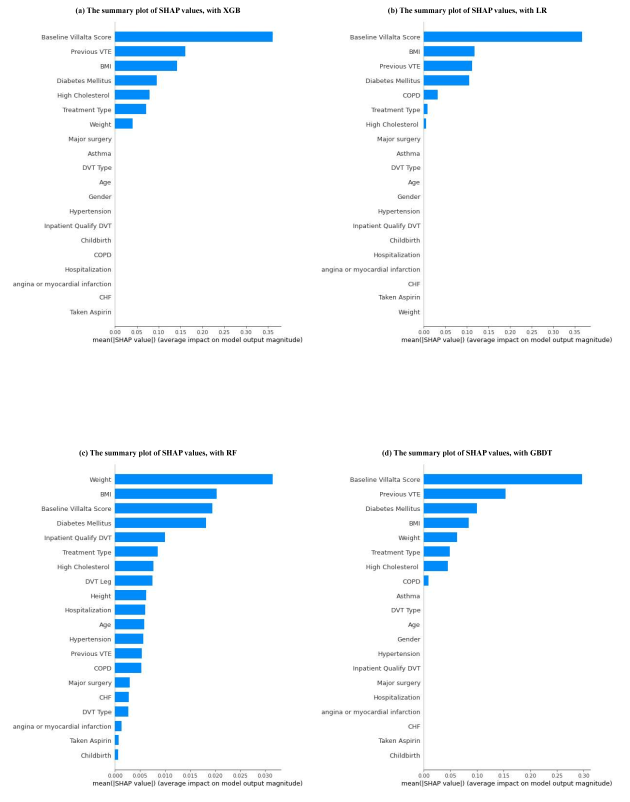


The mean absolute Shapley values are measured as SHAP feature importance. A feature is considered to be “important” if its mean absolute Shapley value is high; a feature is considered to be “unimportant” if its mean absolute Shapley value is low or zero.

Abbreviations: BMI, body mass index; DVT, deep vein thrombosis; VTE, venous thromboembolism; COPD, chronic obstructive pulmonary disease; CHF, congestive heart failure; SHAP, SHapley Additive exPlanations; PTS, post-thrombotic syndrome; RF, random forest; LR, logistic regression; GBDT, gradient boosting decision tree; XGB, extreme gradient boosting.

# Supplementary Figure 10 The feature importance plot of moderate-severe PTS in 24 months


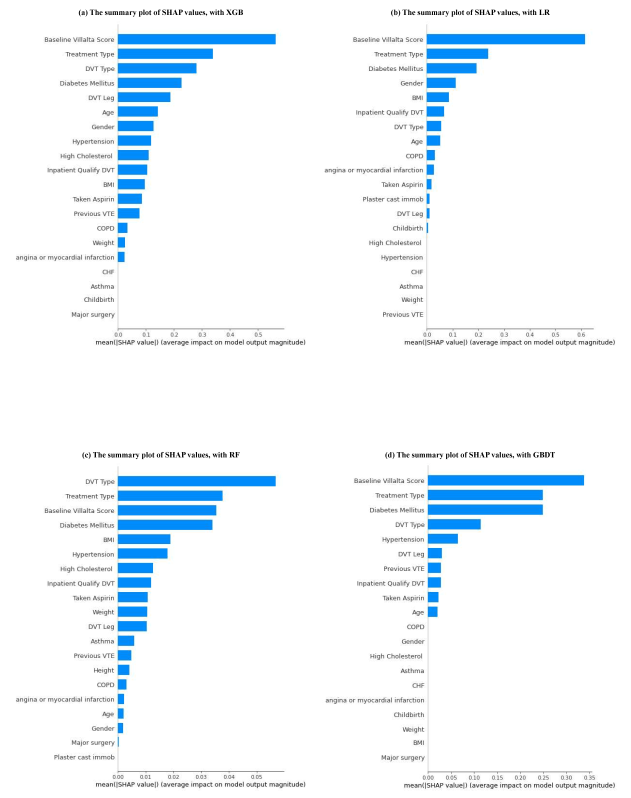


The mean absolute Shapley values are measured as SHAP feature importance. A feature is considered to be “important” if its mean absolute Shapley value is high; a feature is considered to be “unimportant” if its mean absolute Shapley value is low or zero.

Abbreviations: BMI, body mass index; DVT, deep vein thrombosis; VTE, venous thromboembolism; COPD, chronic obstructive pulmonary disease; CHF, congestive heart failure; SHAP, SHapley Additive exPlanations; PTS, post-thrombotic syndrome; RF, random forest; LR, logistic regression; GBDT, gradient boosting decision tree; XGB, extreme gradient boosting.

# Supplementary Figure 11 The feature importance plot of severe PTS in 24 months


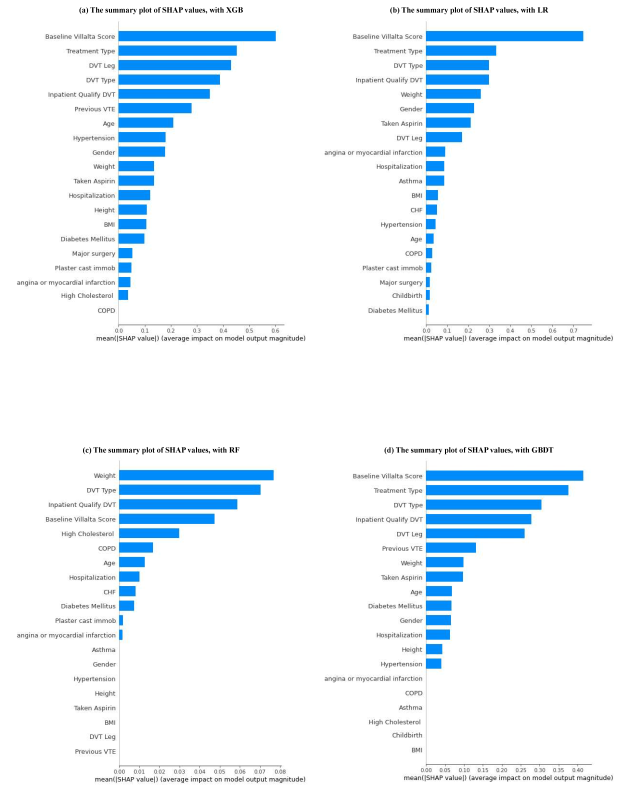


The mean absolute Shapley values are measured as SHAP feature importance. A feature is considered to be “important” if its mean absolute Shapley value is high; a feature is considered to be “unimportant” if its mean absolute Shapley value is low or zero.

Abbreviations: BMI, body mass index; DVT, deep vein thrombosis; VTE, venous thromboembolism; COPD, chronic obstructive pulmonary disease; CHF, congestive heart failure; SHAP, SHapley Additive exPlanations; PTS, post-thrombotic syndrome; RF, random forest; LR, logistic regression; GBDT, gradient boosting decision tree; XGB, extreme gradient boosting.

# Supplementary Figure 12 ROC curves for moderate-severe PTS at 2-year follow-up


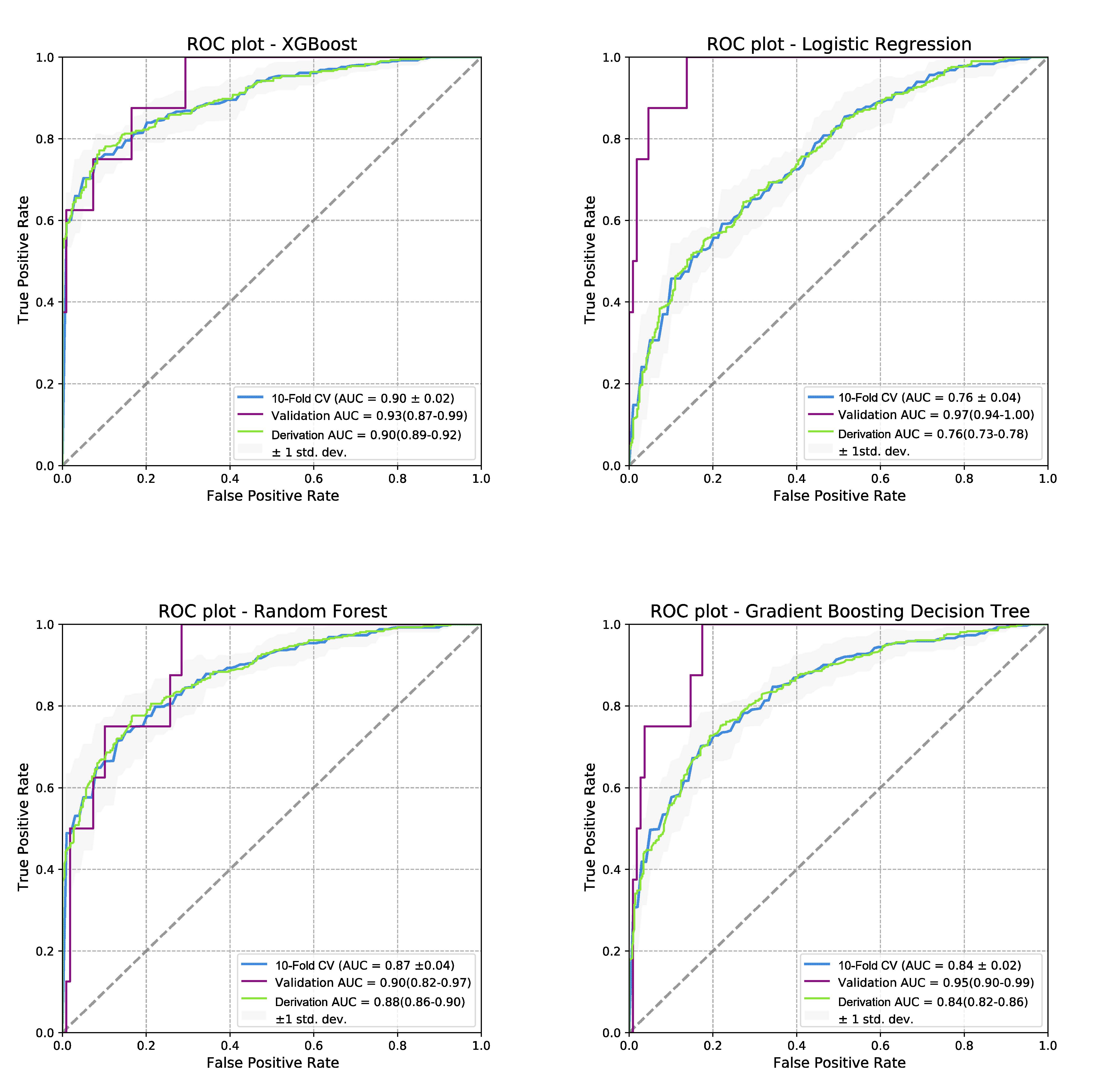


Abbreviations: ROC, receiver operating characteristic curve; PTS, post-thrombotic syndrome; AUC, area under the curve; XGBoost, eXtreme gradient boosting; CV, cross validation.

# Supplementary Figure 13 ROC curves for severe PTS at 2-year follow-up


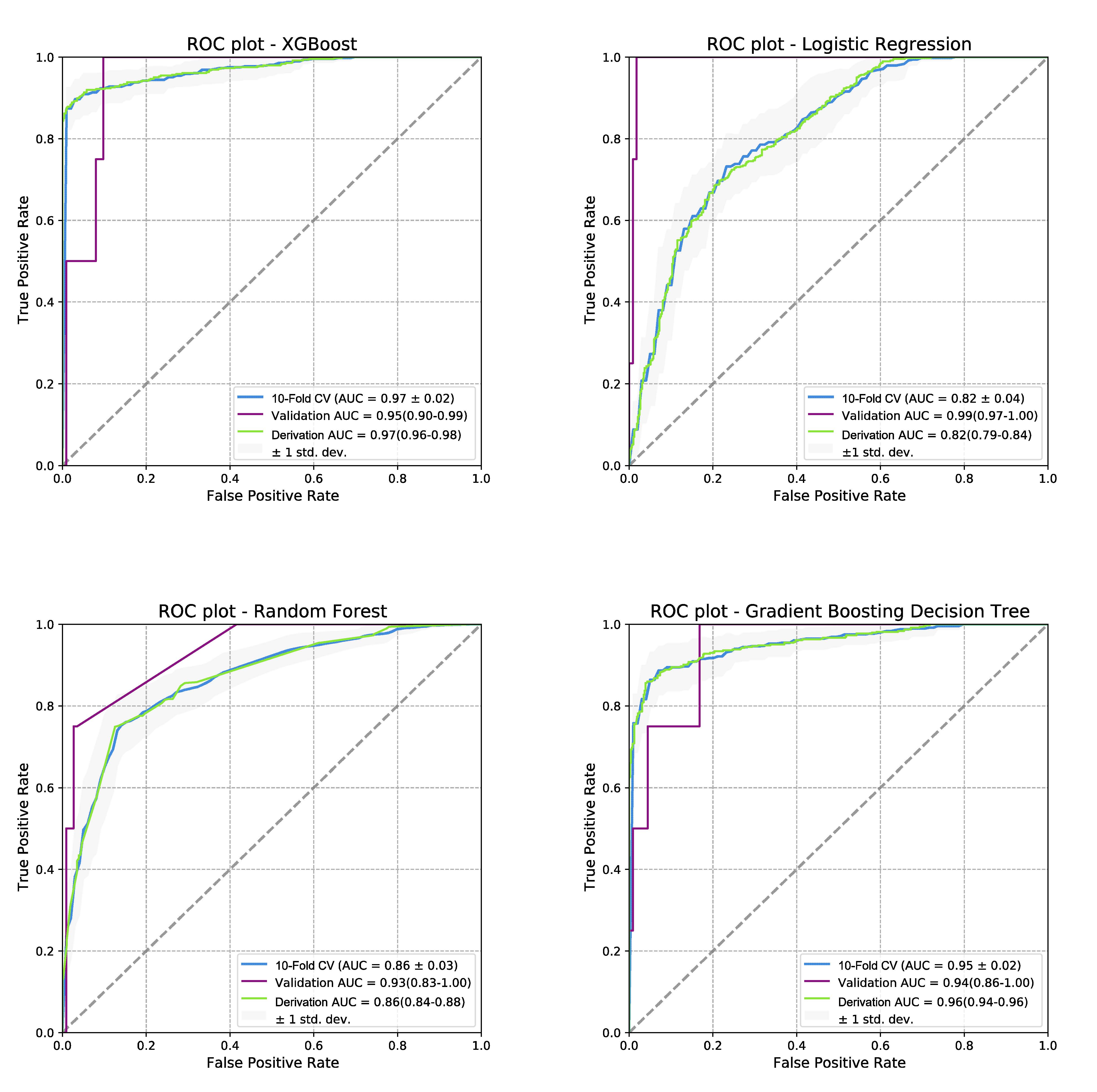


Abbreviations: ROC, receiver operating characteristic curve; PTS, post-thrombotic syndrome; AUC, area under the curve; XGBoost, eXtreme gradient boosting; CV, cross validation.

# Supplementary Figure 14 Calibration curve of ML models built for PTS in external validation cohort


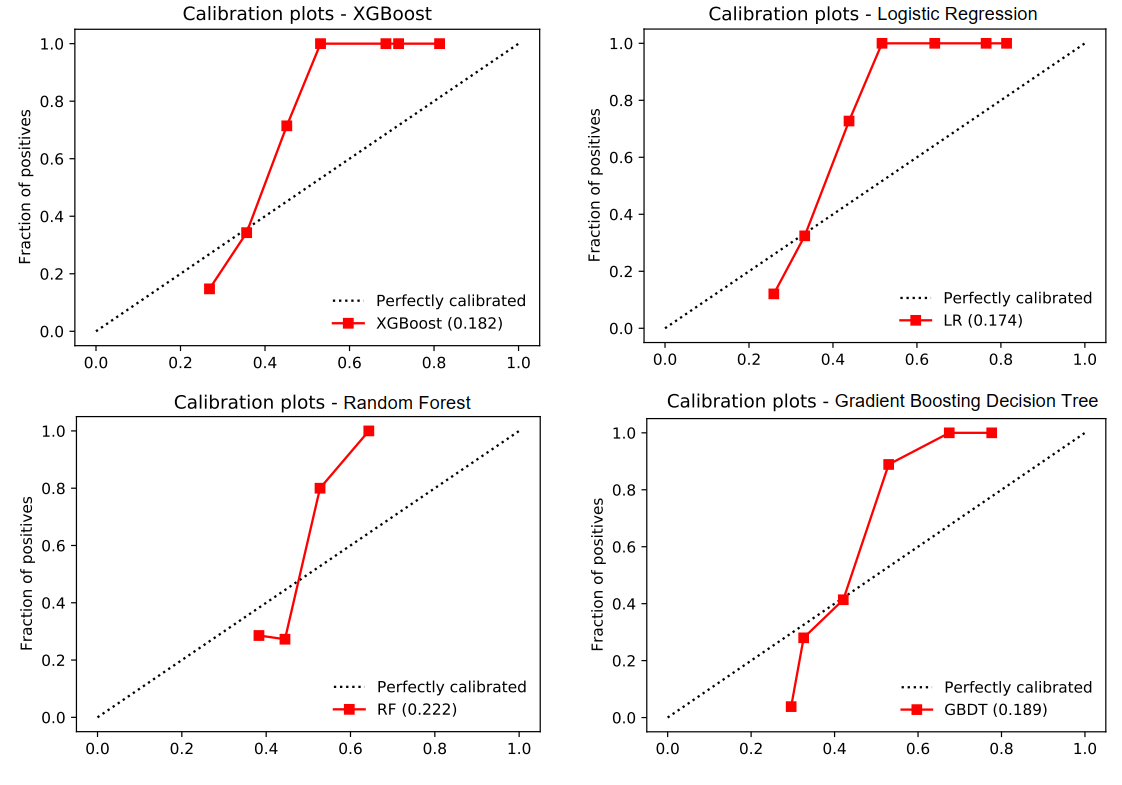


Calibration curve shows the mean predicted probability of outcome against the observed proportion of clinical outcomes.

Abbreviations: ML, machine learning; PTS, post-thrombotic syndrome; XGBoost, eXtreme gradient boosting; GBDT, gradient boosting decision tree; RF, random forest; LR, logistic regression.

# Supplementary Figure 15 Calibration curve of ML models built for PTS in derivation cohort


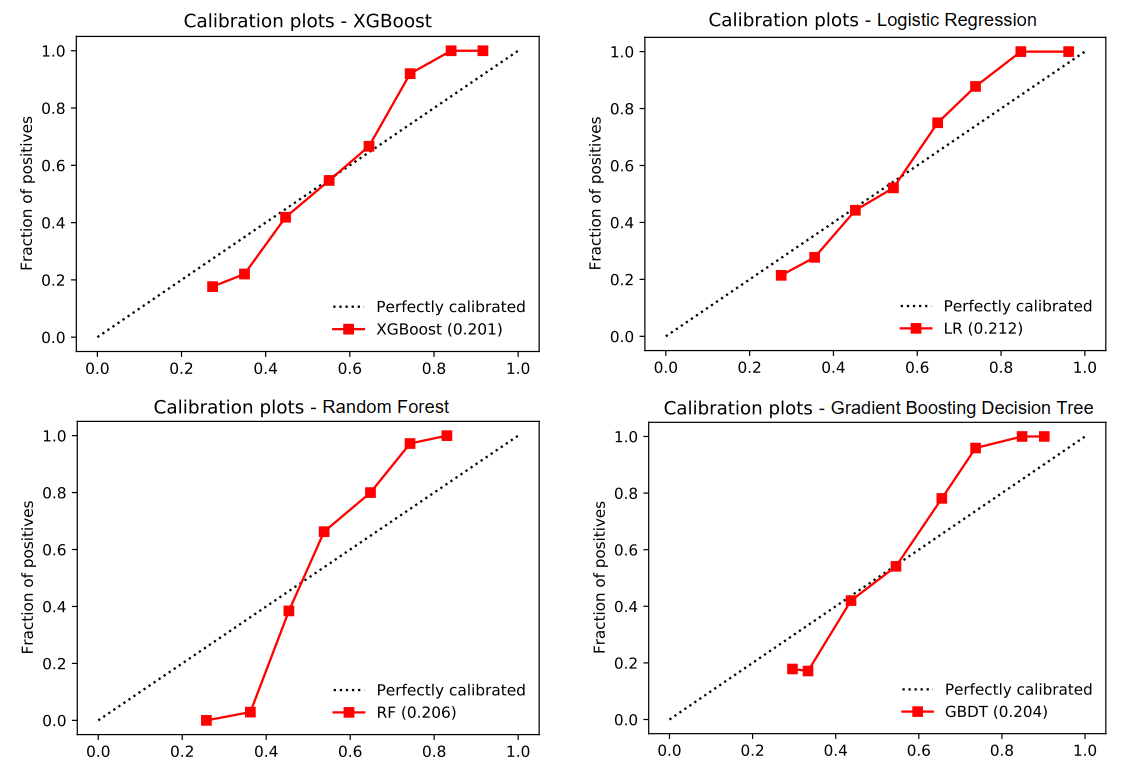


Calibration curve shows the mean predicted probability of outcome against the observed proportion of clinical outcomes.

Abbreviations: ML, machine learning; PTS, post-thrombotic syndrome; XGBoost, eXtreme gradient boosting; GBDT, gradient boosting decision tree; RF, random forest; LR, logistic regression.

# Supplementary Figure 16 Calibration curve of ML models built for moderate-severe PTS in external validation cohort


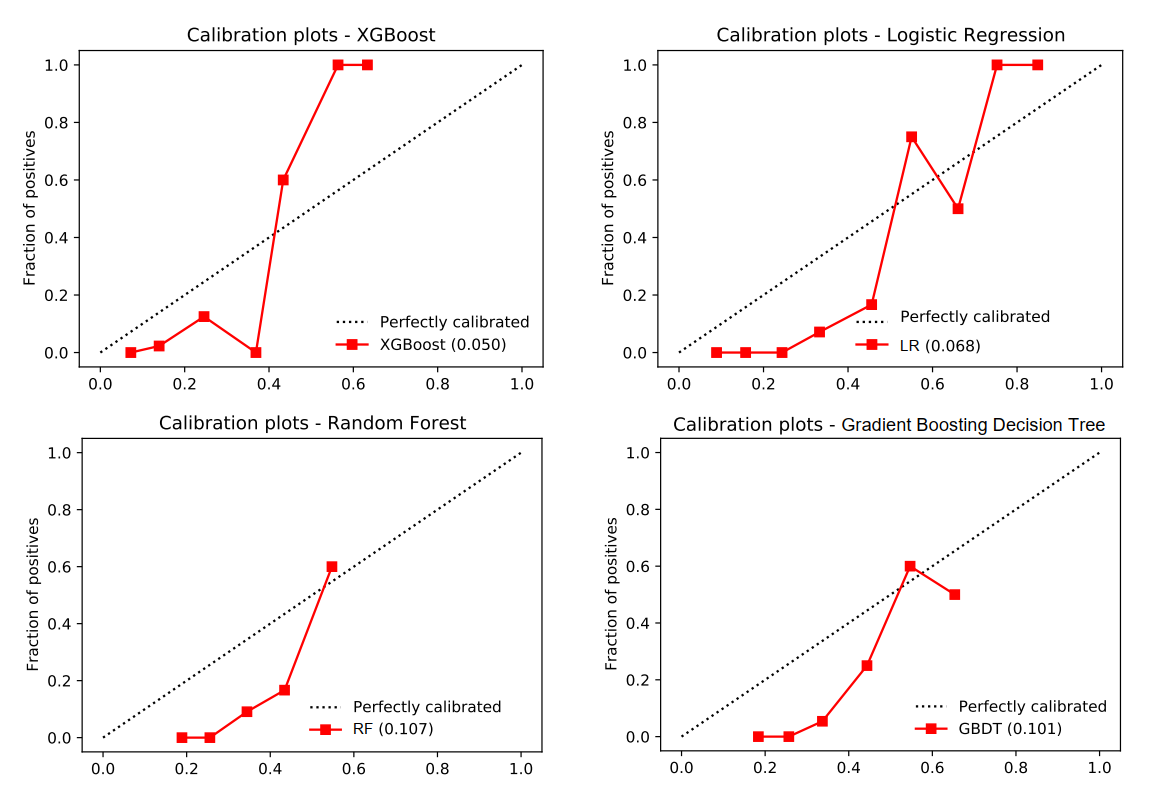


Calibration curve shows the mean predicted probability of outcome against the observed proportion of clinical outcomes.

Abbreviations: ML, machine learning; PTS, post-thrombotic syndrome; XGBoost, eXtreme gradient boosting; GBDT, gradient boosting decision tree; RF, random forest; LR, logistic regression.

# Supplementary Figure 17 Calibration curve of ML models built for moderate-severe PTS in derivation cohort


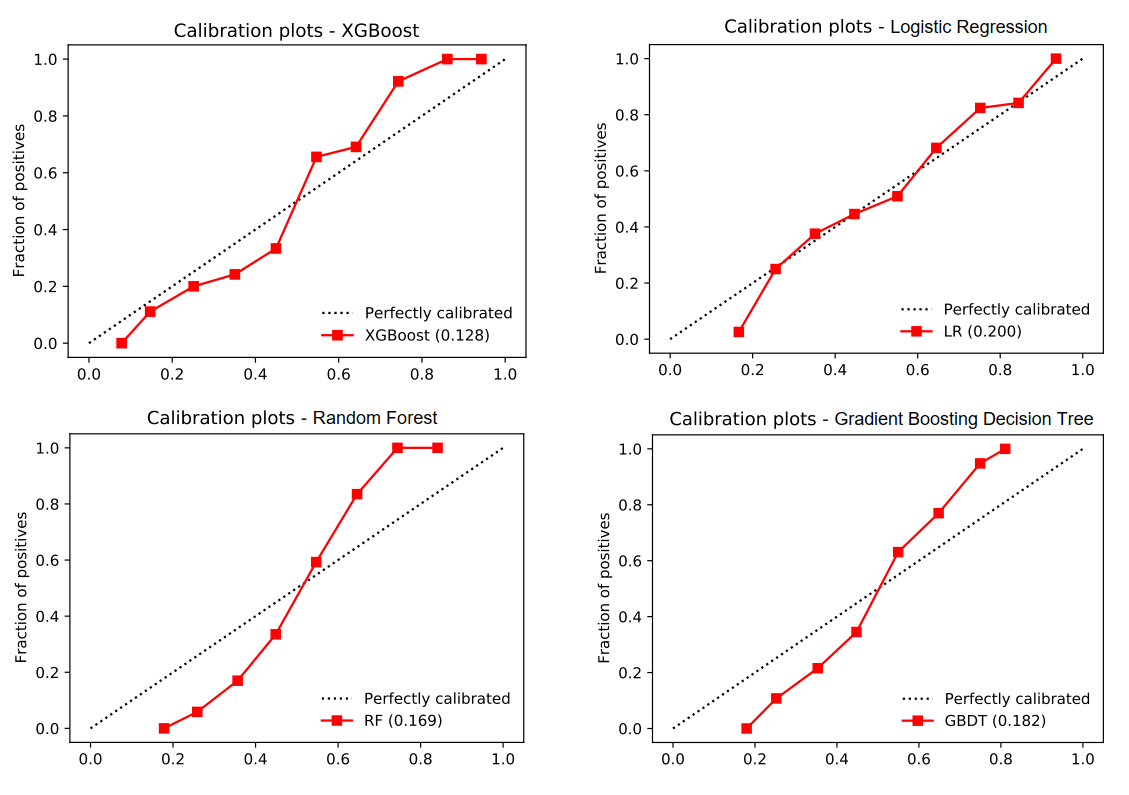


Calibration curve shows the mean predicted probability of outcome against the observed proportion of clinical outcomes.

Abbreviations: ML, machine learning; PTS, post-thrombotic syndrome; XGBoost, eXtreme gradient boosting; GBDT, gradient boosting decision tree; RF, random forest; LR, logistic regression.

# Supplementary Figure 18 Calibration curve of ML models built for severe PTS in external validation cohort


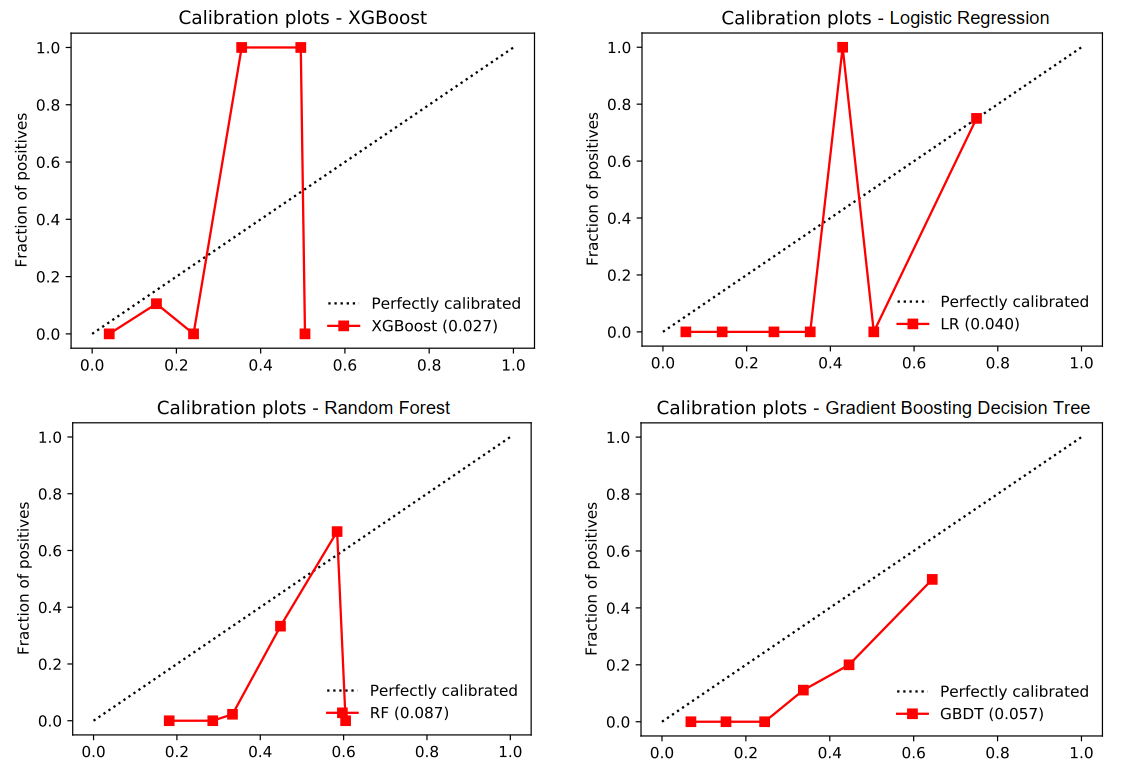


Calibration curve shows the mean predicted probability of outcome against the observed proportion of clinical outcomes.

Abbreviations: ML, machine learning; PTS, post-thrombotic syndrome; XGBoost, eXtreme gradient boosting; GBDT, gradient boosting decision tree; RF, random forest; LR, logistic regression.

# Supplementary Figure 19 Calibration curve of ML models built for severe PTS in derivation cohort


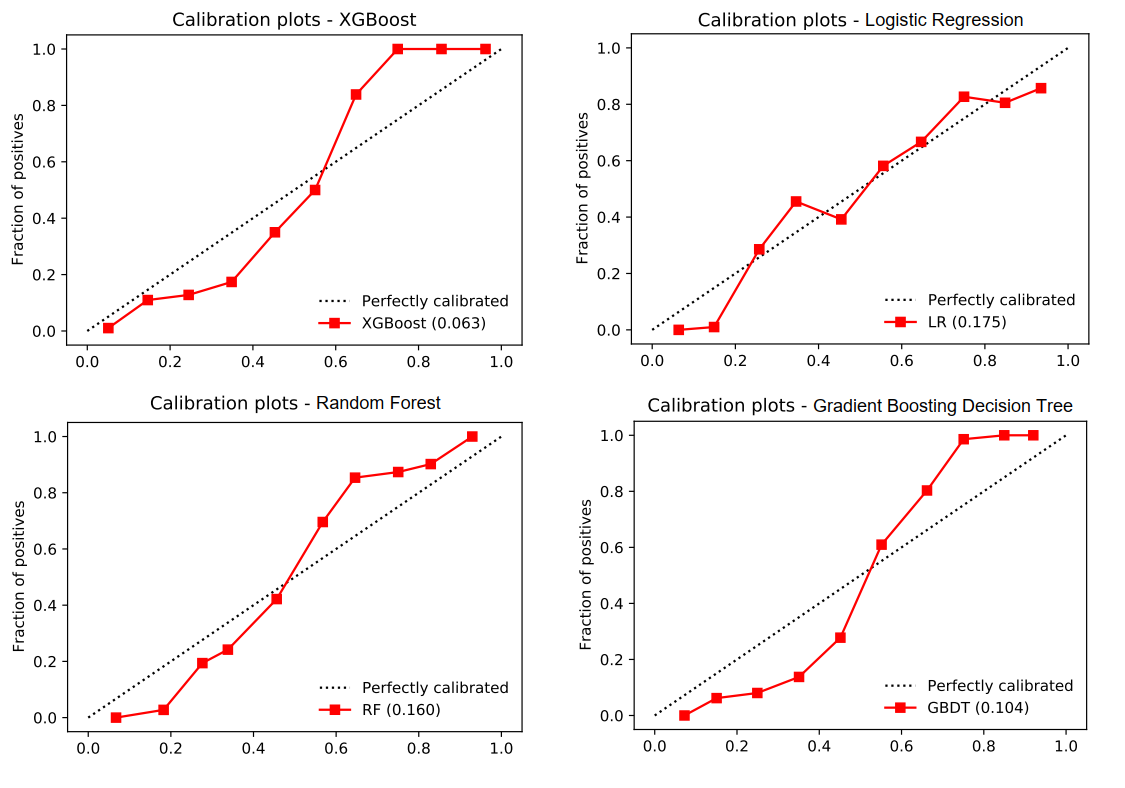


Calibration curve shows the mean predicted probability of outcome against the observed proportion of clinical outcomes.

Abbreviations: ML, machine learning; PTS, post-thrombotic syndrome; XGBoost, eXtreme gradient boosting; GBDT, gradient boosting decision tree; RF, random forest; LR, logistic regression.

# Supplementary Figure 20 AUC of four ML models in predicting different outcomes


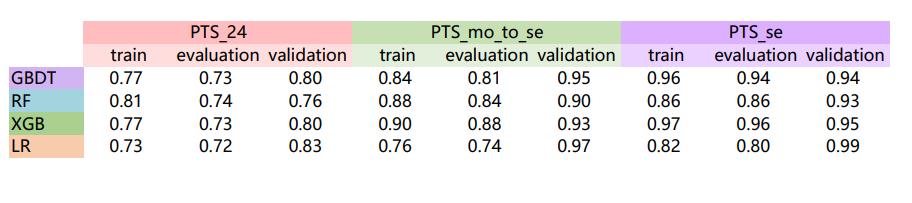


The train column indicates the AUCs in training dataset from derivation cohort. The evaluation column indicates the AUCs using the 10-fold cross validation method in the derivation cohort. The validation column indicates the AUCs in external validation dataset from external validation cohort.

Abbreviations: AUC, area under the curve; ML, machine learning; PTS, post-thrombotic syndrome; GBDT, gradient boosting decision tree; RF, random forest; XGB, eXtreme gradient boosting; LR, logistic regression.

# Supplementary Figure 21 Comparison of performance metrics for four ML models in predicting different outcomes


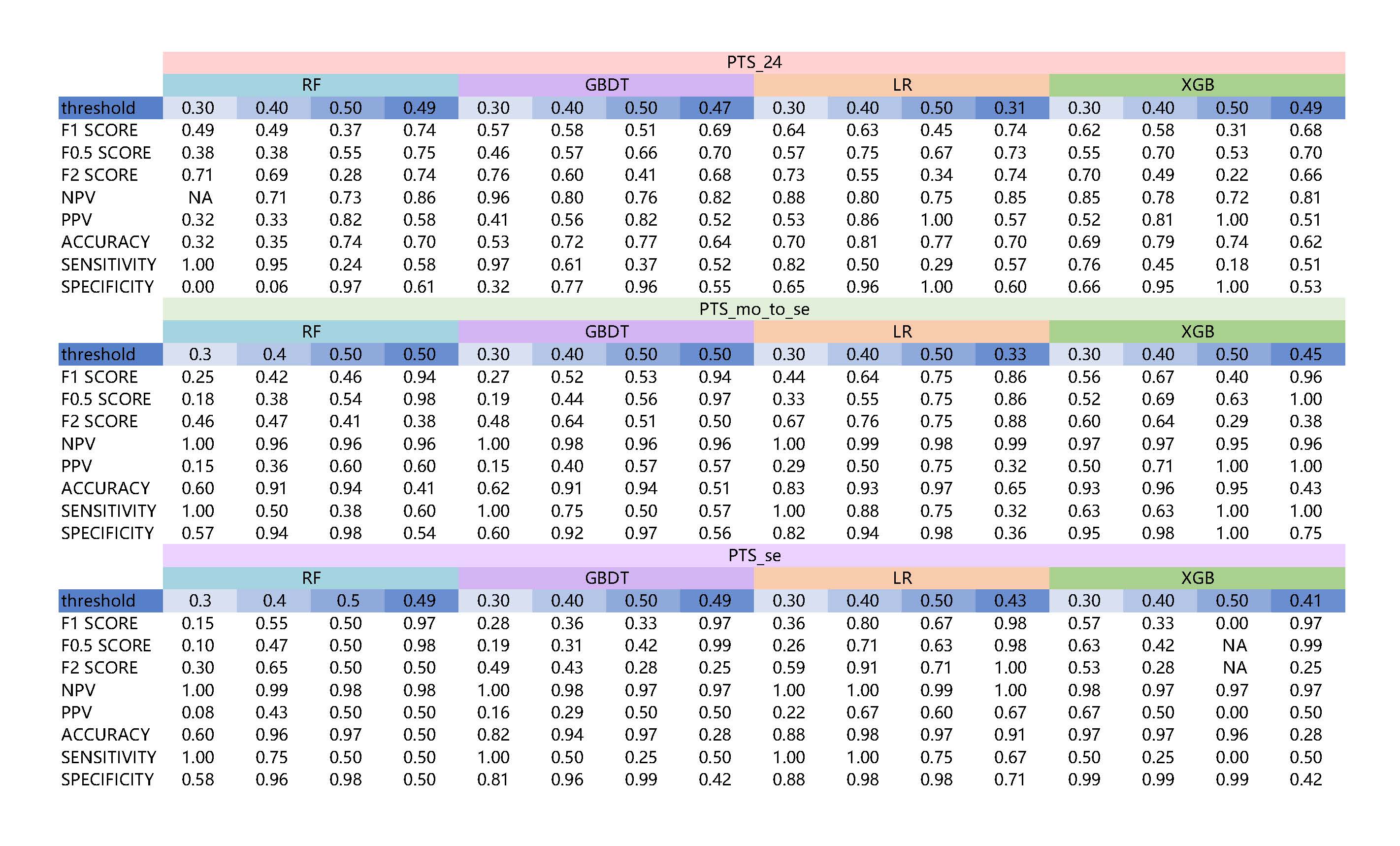


Abbreviations: ML, machine learning; PTS, post-thrombotic syndrome; GBDT, gradient boosting decision tree; RF, random forest; XGB, eXtreme gradient boosting; LR, logistic regression; NPV, negative predictive value; PPV, positive predictive value.

# Supplementary Figure 22 Risk of PTS in 24 months according to deciles of event probability based on four ML models in derivation cohort


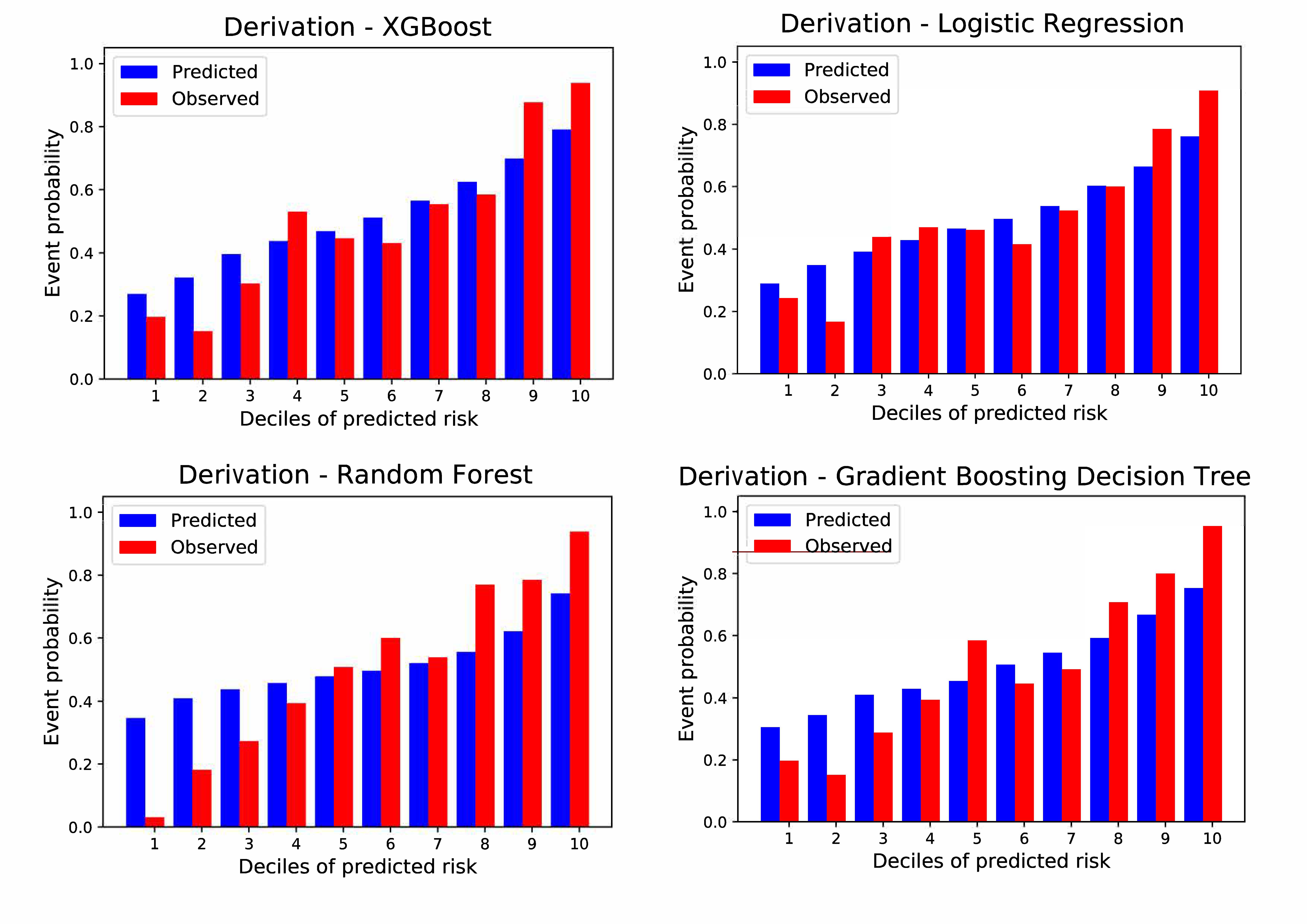


Abbreviations: PTS, post-thrombotic syndrome; ML, machine learning; XGBoost, eXtreme gradient boosting.

# Supplementary Figure 23 Risk of moderate-severe PTS in 24 months according to deciles of event probability based on four ML models in external validation cohort


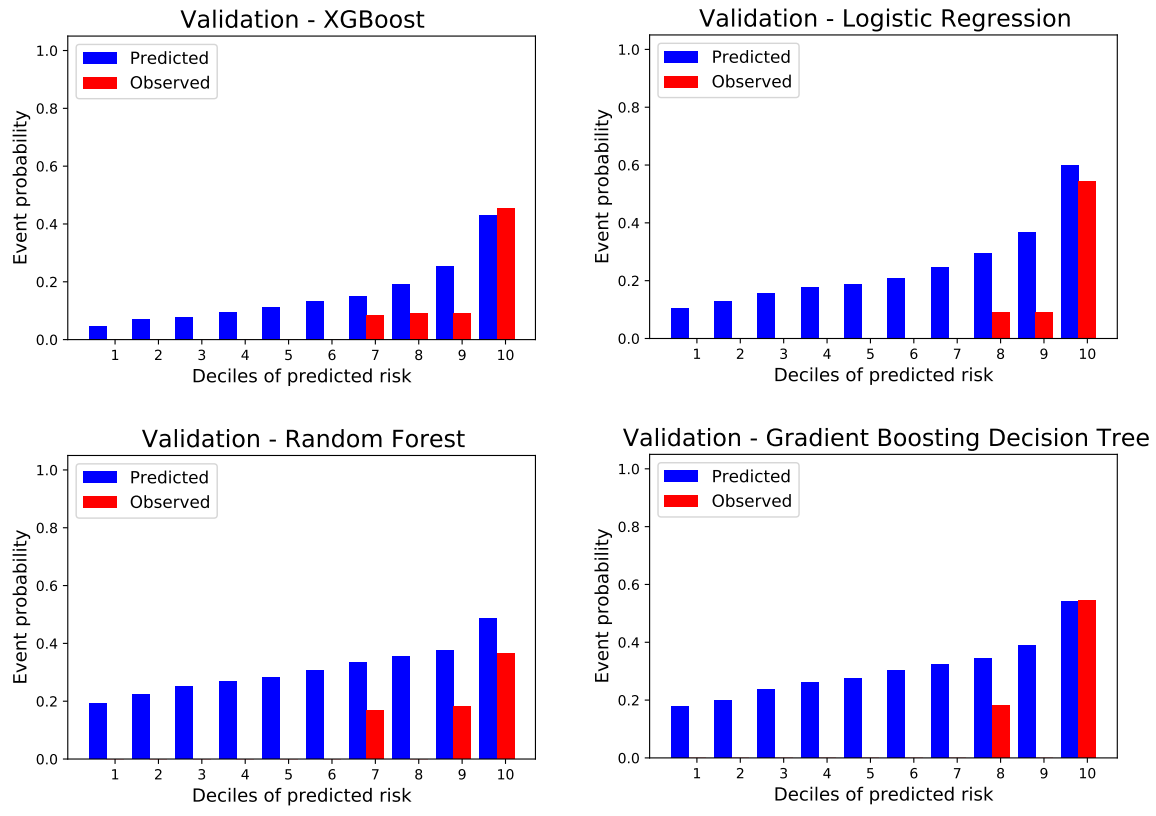


Abbreviations: PTS, post-thrombotic syndrome; ML, machine learning; XGBoost, eXtreme gradient boosting.

# Supplementary Figure 24 Risk of moderate-severe PTS in 24 months according to deciles of event probability based on four ML models in derivation cohort


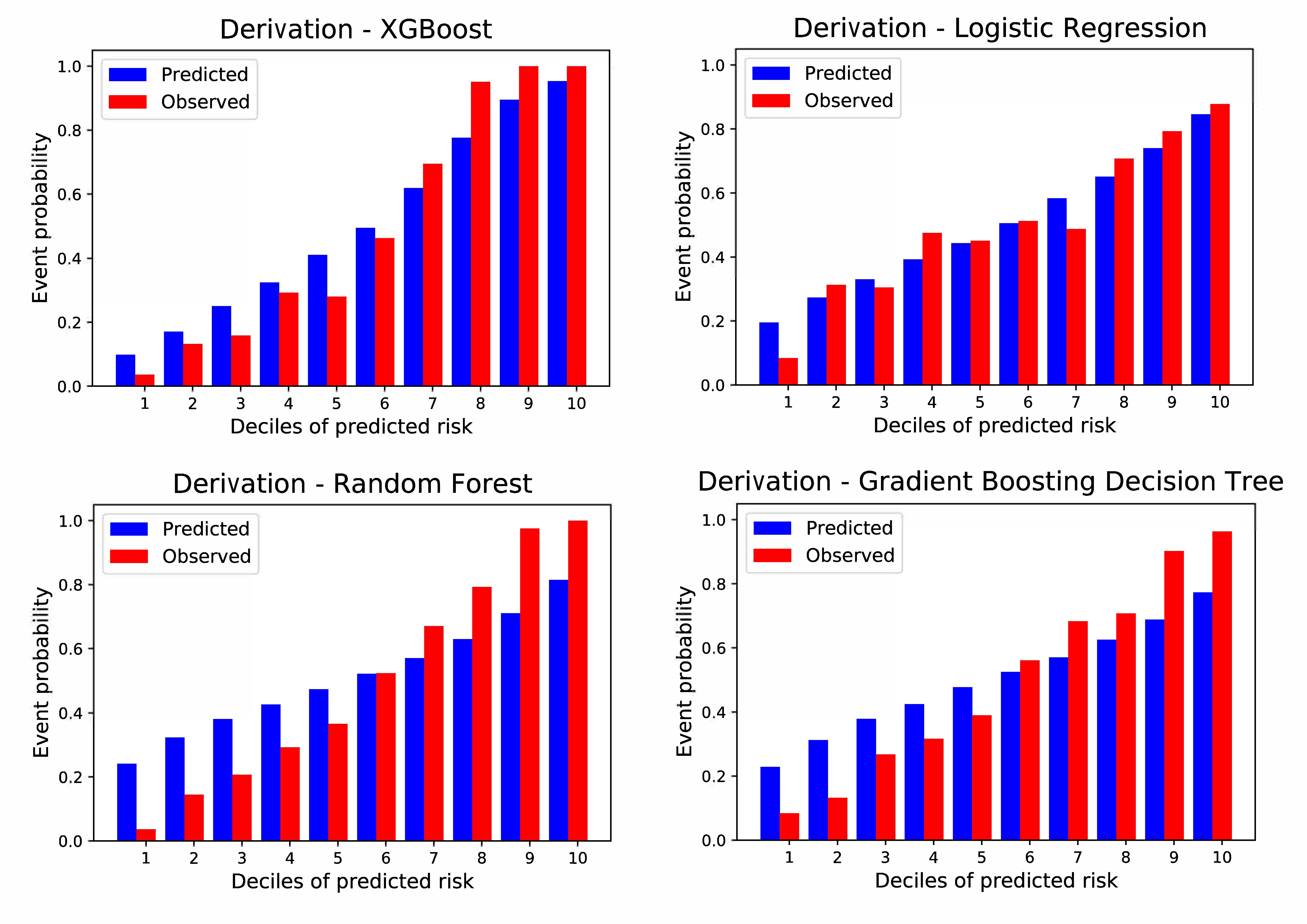


Abbreviations: PTS, post-thrombotic syndrome; ML, machine learning; XGBoost, eXtreme gradient boosting.

# Supplementary Figure 25 Risk of severe PTS in 24 months according to deciles of event probability based on four ML models in external validation cohort


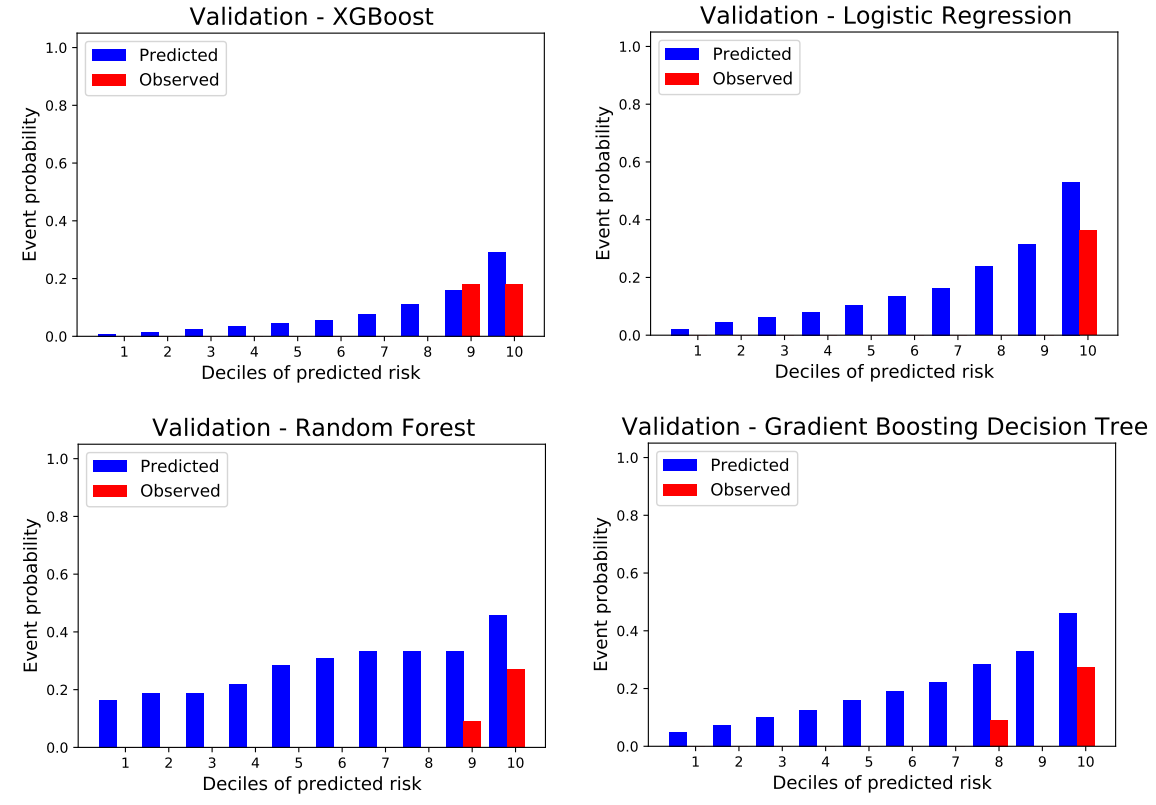


Abbreviations: PTS, post-thrombotic syndrome; ML, machine learning; XGBoost, eXtreme gradient boosting.

# Supplementary Figure 26 Risk of severe PTS in 24 months according to deciles of event probability based on four ML models in derivation cohort


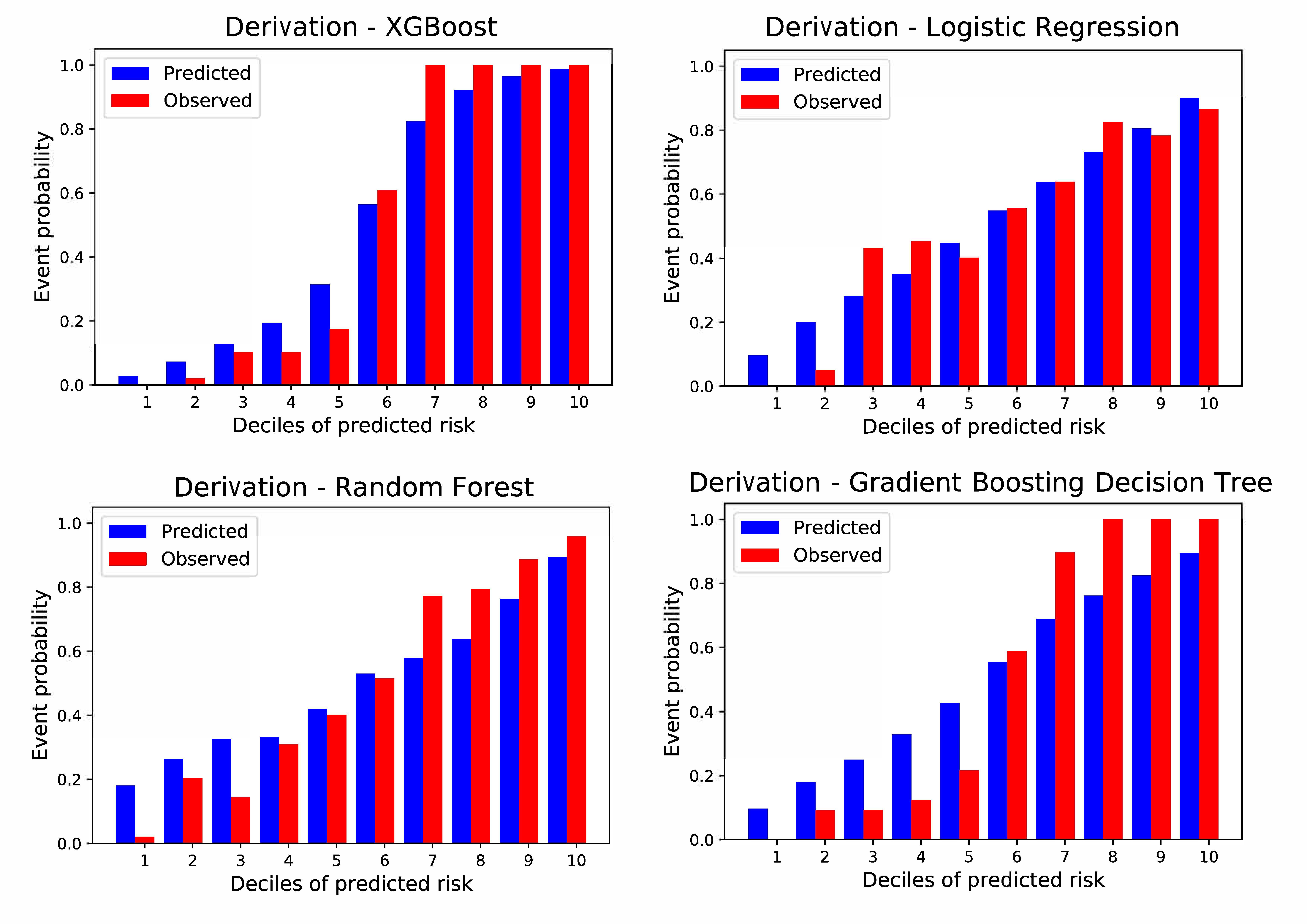


Abbreviations: PTS, post-thrombotic syndrome; ML, machine learning; XGBoost, eXtreme gradient boosting.
